# Profilin2 regulates actin rod assembly in neuronal cells

**DOI:** 10.1038/s41598-021-89397-9

**Published:** 2021-05-13

**Authors:** Lisa Marie Walter, Sebastian Rademacher, Andreas Pich, Peter Claus

**Affiliations:** 1 Institute of Neuroanatomy and Cell Biology, Hannover Medical School, Hannover, Germany; 2 Institute of Toxicology and Core Unit Proteomics, Hannover Medical School, Hannover, Germany; 3 Center for Systems Neuroscience, Hannover, Germany; 4 Present Address: Institute of Biochemistry, Charité-Universitätsmedizin Berlin, Berlin, Germany

**Keywords:** Neurodegeneration, Actin, Motor neuron disease

## Abstract

Nuclear and cytoplasmic actin-cofilin rods are formed transiently under stress conditions to reduce actin filament turnover and ATP hydrolysis. The persistence of these structures has been implicated in disease pathology of several neurological disorders. Recently, the presence of actin rods has been discovered in Spinal Muscular Atrophy (SMA), a neurodegenerative disease affecting predominantly motoneurons leading to muscle weakness and atrophy. This finding underlined the importance of dysregulated actin dynamics in motoneuron loss in SMA. In this study, we characterized actin rods formed in a SMA cell culture model analyzing their composition by LC–MS-based proteomics. Besides actin and cofilin, we identified proteins involved in processes such as ubiquitination, translation or protein folding to be bound to actin rods. This suggests their sequestration to actin rods, thus impairing important cellular functions. Moreover, we showed the involvement of the cytoskeletal protein profilin2 and its upstream effectors RhoA/ROCK in actin rod assembly in SMA. These findings implicate that the formation of actin rods exerts detrimental effects on motoneuron homeostasis by affecting actin dynamics and disturbing essential cellular pathways.

## Introduction

The formation of nuclear and cytoplasmic actin rods is a means to regulate actin dynamics under cellular stress and promotes cell survival^1^. Stressors such as ATP depletion or alteration of the membrane potential induce dephosphorylation of cofilin, which becomes activated and binds to actin in a 1:1 ratio^2–4^. The saturation of filamentous (F)-actin with cofilin stabilizes the filaments and induces their bundling^5,6^. These structures are further linked by the generation of intermolecular disulfide bridges between cofilin monomers^7^.

Three factors have been described to be essential for actin rod assembly: elevated levels of active (dephosphorylated) cofilin, enhanced levels of ADP-actin and a highly oxidative environment^7,8^. Indeed, actin and cofilin are the only proteins found to be present in all stages of actin rod formation^9^. Transient actin rod formation is beneficial as it enables storage of actin, thereby slowing actin filament turnover and ATP hydrolysis^1^. However, a pathological stabilization and persistence of actin rods has been identified in several neurodegenerative diseases such as Alzheimer or Huntington’s disease in which rods sequester actin and cofilin, thereby perturbing actin dynamics and putatively blocking intracellular trafficking causing synaptic loss^8,10,11^. Recently, the presence of nuclear and cytoplasmic actin rods has been described in cellular and mouse models of Spinal Muscular Atrophy (SMA)^12^, a neuromuscular disease in children caused by a lack of the Survival of Motoneuron (SMN) protein^13,14^. Indeed, all three criteria for actin rod formation are fulfilled in SMA. Disruption of mitochondrial homeostasis impairs ATP production and enhances the generation of reactive oxygen species (ROS)^15–17^. Moreover, hypophosphorylated cofilin is associated with a dysregulation of the RhoA/RhoA-associated coiled-coil kinase (ROCK) pathway^18^. Interestingly, actin rods induced by a lack of SMN are decorated with a C-terminal proteolytic fragment of the semaphorin surface receptor plexinD1 (PLXND1)^12^. This protein induces cell death by activating the mitochondrial apoptosis pathway^19^. Thus, it was hypothesized that actin rods function as a sink to sequester PLXND1 and prevent mitochondrial translocation^12^.

In this study, we analyzed the protein composition of purified actin rods under SMA conditions by LC–MS-based proteomics to identify putative signaling pathways associated with actin rod formation. Besides a variety of proteins involved in ATP-consuming processes important for cellular homeostasis, we identified the cytoskeletal protein profilin2 to be bound to actin rods. Further analysis revealed its involvement in actin rod assembly in SMA which is partially mediated by its phosphorylation at serine residue 137. In line with that, we showed an involvement of its upstream kinase ROCK in actin rod formation independent from cofilin hypophosphorylation. The results of our study underline the contribution of dysregulated actin dynamics as a pathogenic pathway in SMA.

## Results

### Neuronal profilin2 is a component of actin rods in SMA

**Figure 1:**
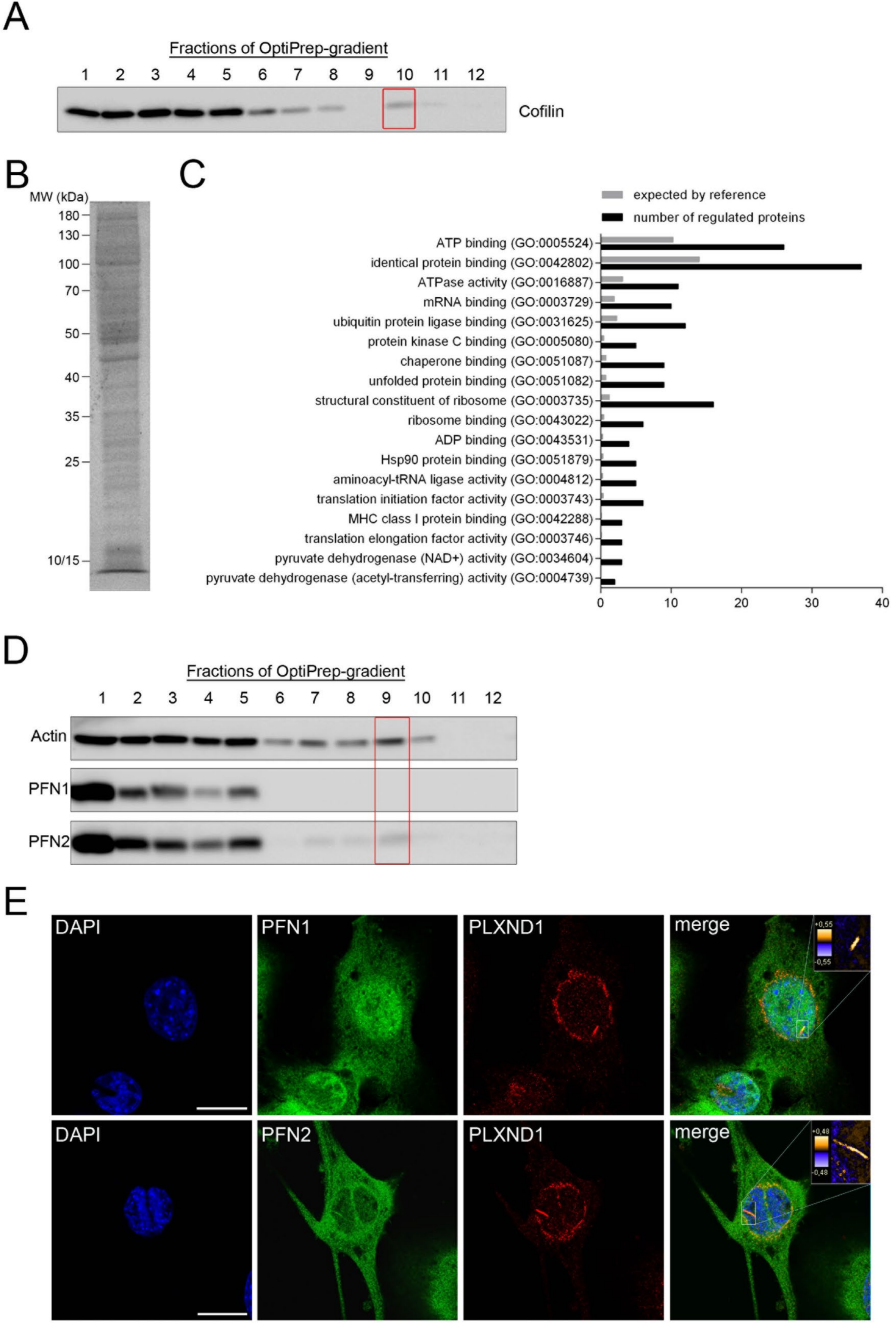
Profilin2 is a component of actin rods formed in SMA. Actin rods were enriched according to earlier studies^9,12^. Cells were treated with siSmn and the lysate was separated on a 10%/15%-Optiprep gradient. (**A**) Fractions from the gradient were analyzed by Western blot. Actin rods (red box at fraction 10) were distinguished from cytoplasmic proteins (Fractions 1–5) by incubation of the blot with an anti-cofilin antibody. (**B**) 12.5% Coomassie-stained SDS-PAGE of the concentrated fraction 10 of (A). Labeled bands were analyzed by LC–MS. (**C**) Enrichment of proteins after identification by mass-spectrometry and classified by their molecular function using the PANTHER overrepresentation test (gene ontology database) and the mouse genome as reference. (**D**) Western blot of fractions after separation of SMN-depleted cell lysate on a 10%/40%-Optiprep gradient. Rods were detected by using an antibody against actin. In addition, fractions were probed for profilin1 (PFN1) and profilin2 (PFN2). (**E**) Confocal images of siSmn-treated NSC34 cells co-stained for PLXND1 and PFN1 or 2. Co-localization was analyzed by measuring the product of the differences of the mean (PDM) and those data are represented in the merged color-coded images. The PDM values represent a quantitative measure of colocalization and their numeric values are demonstrated by false colors (insets). DAPI was used as nuclear counterstaining. Scale bar: 20 µm.

Nuclear and cytoplasmic actin rod formation in SMA has recently been described by our group^12^. These rods show the same characteristics as actin rods observed in other neurodegenerative diseases^4,7,12^. However, little is known about the composition of the actin rods in SMA. Until now, only the main components actin and cofilin as well as the carboxy terminal fragment of the surface receptor PLXND1 have been identified as factors differentially interacting with actin rods under SMA conditions^12^. Thus, we performed LC–MS based proteomics of enriched actin rods to detect interacting proteins that may be involved in their formation (Fig. 1). We used motoneuron-like NSC34 cells and transfected them with siRNA against murine *Smn* to induce actin rod assembly^12,20^. After three days of differentiation, cells were harvested and actin rods were enriched on a 10%/40%-OptiPrep gradient (Fig. 1A). Actin rods were collected at the interphase, concentrated and separated by SDS-PAGE (Fig. 1B). Proteins were in-gel digested with trypsin and analyzed by LC–MS. In total, 2522 different proteins were identified (Supplementary Table 1), of which actin and cofilin were among the most abundant ones. 517 of the identified proteins were further analyzed by gene ontology (GO) classification. For enrichment, the PANTHER overrepresentation test was used with the mouse genome as reference^21–23^ (Fig. 1C). Many of the proteins identified in the actin rod enriched fraction are involved in ATP-consuming processes such as translation, ubiquitination, phosphorylation or refolding of denatured proteins^24–27^ (Fig. 1C). This finding suggested a more global role of actin rod formation in SMN-lacking cells.

Interestingly, LC–MS analysis revealed the association of actin rods with profilin (PFN), another actin-binding protein which regulates actin polymerization and was shown to directly interact with the SMN protein (Supplementary Table 1)^18,28–30^. Although profilin1, the ubiquitously expressed isoform, is more abundant in NSC34 cells, we identified the neuronal profilin2 in the actin rod enriched fraction (Supplementary Table 2). We validated this finding by Western blot in which only profilin2 but not profilin1 showed a signal in the actin rod fraction (Fig. 1D). However, confocal microscopy of SMN knock-down cells stained with profilin1 or 2 antibodies and PLXND1 antibody as control for actin rod formation revealed co-localization of both profilin isoforms with actin rods (Fig. 1E). Nonetheless, based on the lack of signal in the LC–MS analysis as well as on Western blots (Supplementary Table 1, Fig. 1D), we propose that the amount of profilin2 bound to actin rods exceeds that of profilin1.

### Profilin2 is involved in actin rod formation

**Figure 2:**
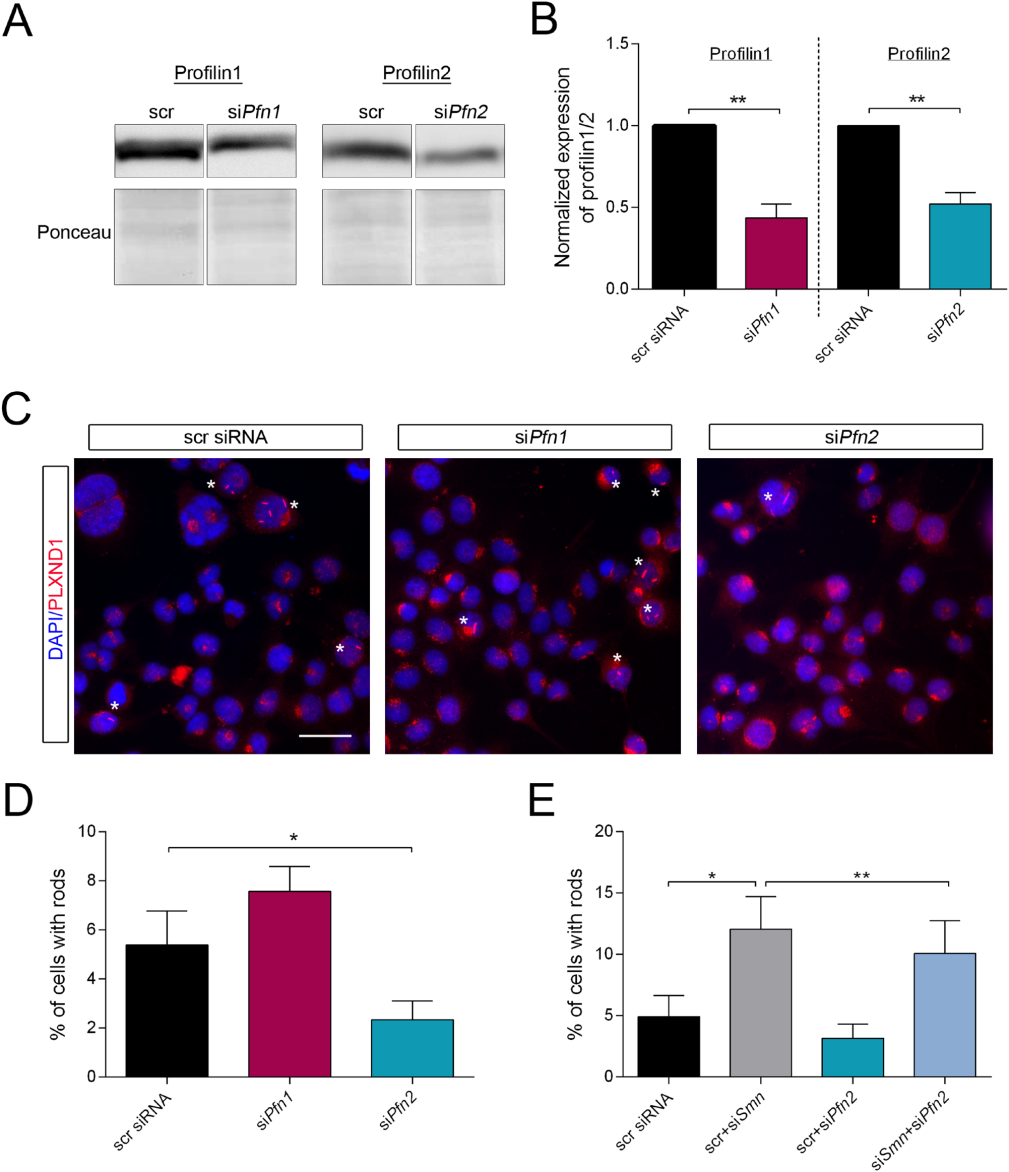
Profilin2 but not profilin1 is involved in actin rod formation. (**A**) Representative Western blots of cells treated with si*Pfn1* or si*Pfn2*. Ponceau was used as loading control. (**B**) Quantification of PFN1 and PFN2 signal normalized to total protein (mean ± SEM, n = 3, unpaired two-tailed t-test, **p < 0.01). (**C**) Immunofluorescence detection of actin rods by PLXND1 antibody in NSC34 cells treated with scr siRNA, si*Pfn1* or si*Pfn2*. Cells with actin rods are labeled with an asterisk. DAPI was used as nuclear counterstaining. Scale bar: 20 µm. (**D**) Quantification of actin rods in cells treated with scr siRNA, si*Pfn1* or si*Pfn2* (mean ± SEM, n = 5 independent biological replicates, (one-way ANOVA, Dunnett’s multiple comparison test, *p < 0.05). The following ranges represent different numbers of cells counted per condition as stated on the x-axis. Cell number counted for each replicate number was: #1: 374–503 cells; #2: 525–778 cells; #3: 406–612 cells; #4: 742–809 cells; #5: 648–698 cells. (**E**) Quantification of cells with rods after co-transfection with scr, si*Smn* and si*Pfn2* in combinations as indicated (mean ± SEM, n = 4 independent biological replicates, one-way ANOVA, Tukey’s multiple comparisons test, *p < 0.05, **p < 0.01). The following ranges represent different numbers of cells counted per condition as stated on the x-axis. Cell number counted for each replicate number was: #1: 510–561 cells; #2: 606–777 cells; #3: 691–1024 cells; #4: 573–838 cells.

Two possible, non-exclusive models could explain the presence of profilin1 and 2 on actin rods. Either both proteins are sequestered by actin rods as seen for actin and cofilin or profilin1 and/or 2 contribute to actin rod formation in SMA. We addressed this question by generating siRNA against murine *Pfn1* and *Pfn2* and assessed their impact on actin rod assembly (Fig. 2). Both siRNAs led to a protein reduction of about 50% (Fig. 2A,B). Quantification of cells with actin rods was performed by staining for PLXND1 (Fig. 2C,D). Remarkably, we detected a significant reduction of actin rod formation by lowering the levels of profilin2 only (Fig. 2D). This supports the hypothesis that the amount of profilin1 on actin rods is just marginal and does not regulate rod assembly. Accordingly, we focused on profilin2 in subsequent experiments. In a co-knock-down experiment, we tested the effect of profilin2 reduction in a SMA background (Fig. 2E). Lowering the amount of SMN induced actin rod formation which was decreased by simultaneously reducing the profilin2 levels. These findings indicate a modulatory function of profilin2 in actin rod assembly in SMA rather than a simple sequestering effect.

### Phosphorylation of profilin2 additionally modulates actin rod formation in SMA

**Figure 3:**
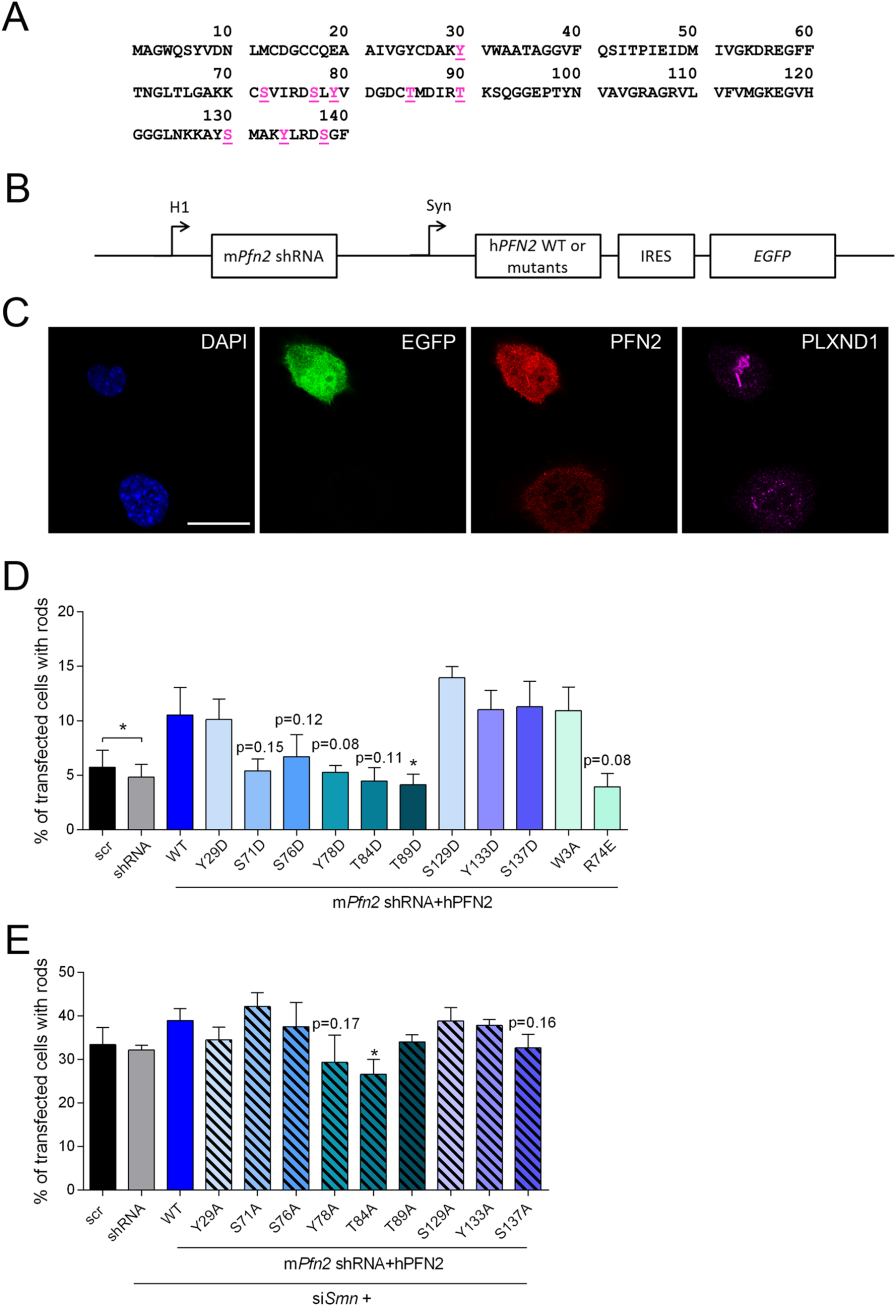
Single-site phosphorylation of profilin2 shows only minor effects on rod formation. (**A**) Primary structure of human PFN2 with putative phosphorylation sites (magenta). (**B**) Schematic drawing of a bicistronic plasmid for knock-down of endogenous mPFN2 and overexpression of hPFN2 WT and mutants, respectively. The EGFP gene was molecularly cloned under the control of an IRES sequence to function as a transfection control. (**C**) Confocal images of NSC34 cells transfected with the plasmid of (**B**) comprising the coding sequence of hPFN2 WT. Cells were stained with PFN2 and PLXND1 antibody. DAPI was used as nuclear staining. Scale bar: 20 µm. (**D**) Quantification of rod-containing cells transfected with the bicistronic plasmids comprising the coding sequences for *hPFN2* phospho-mimetics (D-mutants) (mean ± SEM, n = 5 independent biological replicates, paired two-tailed t-tests in comparison to WT if not otherwise indicated, *p < 0.05). The following cell numbers were counted for each replicate: #1: 89–214 cells; #2: 108–238 cells; #3: 77–249 cells; #4: 95–235 cells; #5. 115–225 cells. (**E**) Quantification of cells with rods after co-transfection with si*Smn* and the bicistronic plasmids comprising the coding sequences of *hPFN2* phospho-mutants (A-mutants) (mean ± SEM, n = 4 independent biological replicates, paired two-tailed t-tests in comparison to WT, *p < 0.05). The following cell numbers were counted for each replicate: #1: 75–144 cells; #2: 81–154 cells; #3: 90–179 cells; #4: 102–146 cells.

Profilin2 is hyperphosphorylated in SMA conditions which is at least partially mediated by a dysregulation of the ROCK kinase^18^. The phosphorylation of profilin2 at specific amino acid residues may alter its binding properties contributing to actin rod formation in SMA (Fig. 2E). As the respective phospho-sites of profilin2 have not been determined yet, we performed an in silico analysis using the NetPhos 3.1 platform to identify putative phospho-sites of human profilin2 (Fig. 3A). The algorithms use an artificial neural network to predict generic and kinase-specific phosphorylation sites^31,32^. We selected nine phospho-sites based on their amino acid residue and their relative position in the primary structure. One of the phospho-sites, S137, was already described to be targeted in profilin1 by ROCK1^33^. Next, we exchanged these residues for aspartate or alanine to mimic phosphorylation (D-mutants) or the non-phosphorylated state (A-mutants) at the respective sites. Using a bicistronic vector comprising shRNA against murine profilin2 and the coding sequences for the human profilin2 mutants allowed us to express the phospho-mutants by simultaneously reducing the amount of endogenous protein (Fig. 3B, Supplementary Fig. 1). Additionally, EGFP was molecularly cloned under the control of an IRES sequence behind the coding sequence of profilin2 to serve as transfection control. Expression of the phospho-mutants was checked by immunofluorescence staining and Western blot (Fig. 3C, Supplementary Fig. 2). First, we overexpressed profilin2 phospho-mimetics to identify phospho-sites with an additive effect on actin rod formation induced by overexpression of profilin2 (Fig. 3D). Besides the D-mutants, we included two profilin2 mutants with known effects on their binding to interaction partners such as actin, phosphatidylinositol (4,5)-bisphosphate (PIP_2_) and proteins with poly-L-proline (PLP) stretches^34–38^. Profilin2 W3A is a mutant deficient in PLP-binding, while profilin2 R74E shows a disrupted interaction with actin^39^. Interestingly, profilin2 S71D, S76D, Y78D, T84D and R74E showed a tendency towards decreased actin rod formation compared to WT. Only profilin2 T89D revealed a significant reduction in actin rod assembly which is possibly caused by its low expression level (Fig. 3D, Supplementary Fig. 2). In contrast, profilin2 Y133D and S137D did not affect actin rod formation compared to WT, although their expression levels were lower. This suggests altered properties of these mutants promoting the assembly of actin rods which may become important in SMA. Thus, we tested the effect of overexpressing profilin2 A-mutants in a SMA background (Fig. 3E). In contrast to our prior findings (Fig. 2E), we did not detect any effect on actin rod formation of profilin2 knock-down in SMN-depleted cells. This may be caused by differences in cell counting strategies. In this experiment, we considered only cells expressing GFP, while the prior experiments lacked a transfection control and thus all cells were counted. Nonetheless, this experimental setup is suitable for the evaluation of the impact of profilin2 phosphorylation on actin rod assembly in SMA, as overexpression of the WT still showed an enhanced number of cells with actin rods. We detected a significant reduction of actin rod assembly in the presence of profilin2 T84A. However, this mutant was also expressed at low levels (Supplementary Fig. 2). In contrast, profilin2 Y78A and S137A were expressed at WT-like levels, but showed a tendency towards reduced actin rod assembly (Fig. 3E, Supplementary Fig. 2). As profilin2 Y78D had also a rod-reducing effect in the prior experiment (Fig. 3D), we assume that exchanging the amino acid residue at this position impairs the protein’s function to induce actin rod assembly which is independent from the putative phosphorylation of this site. However, mimicking the non-phosphorylated state at profilin2 S137 tended to reduce the number of cells with actin rods indicating the contribution of this phospho-site in actin rod formation in SMA.

### RhoA-ROCK signaling contributes to rod formation in SMA

**Figure 4:**
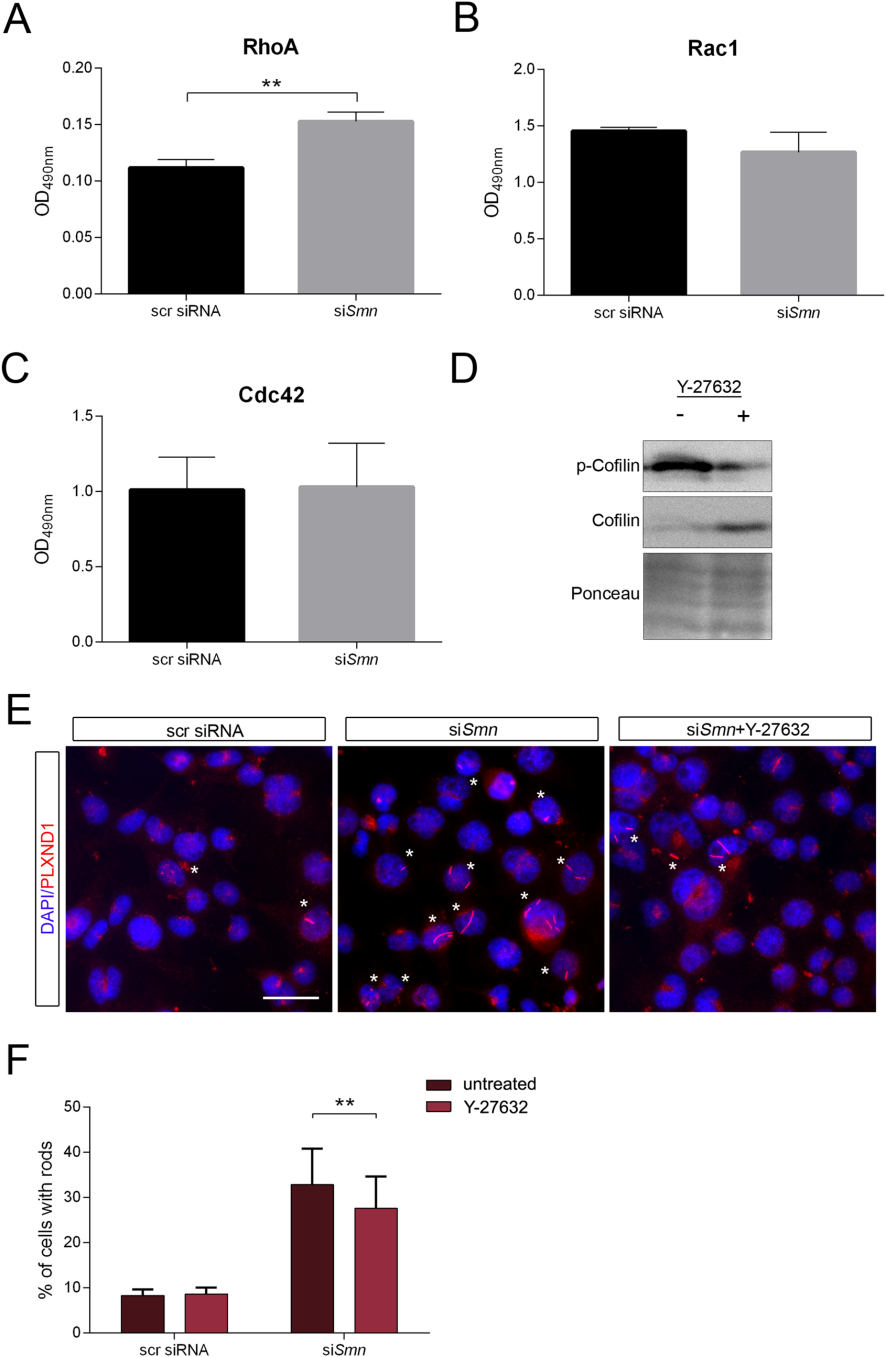
RhoA-ROCK axis is involved in actin rod formation in SMN knock-down cells**.** Cells were treated with scr siRNA or si*Smn* and differentiated for three days. Activity of small G-proteins [(**A**) RhoA, (**B**) Rac1, (**C**) Cdc42] was measured by using G-LISA Activation Assays (Cytoskeleton) (mean ± SEM, n = 3, paired two-tailed t-test, **p < 0.01). (**D**) Efficacy of the ROCK inhibitor Y-27632 (50 µM for three days) shown by Western blot of p-cofilin and cofilin. Ponceau staining was used as loading control. (**E**) Representative immunofluorescence images of actin rods in NSC34 cells treated with scr siRNA, si*Smn* or si*Smn* + Y27632. Actin rods were detected by PLXND1 antibody and labeled with an asterisk. DAPI was used as nuclear counterstaining. Scale bar: 20 µm. (**F**) Quantification of actin rods in cells treated with scr or *siSmn* with and without Y27632. (Mean ± SEM, n = 4 independent biological replicates, two-way ANOVA, Sidak’s multiple comparisons test, **p < 0.01). The following cell numbers were counted for each replicate: #1: 388–737 cells; #2: 492–704 cells; #3: 680–741 cells; #4: 719–984 cells.

The RhoA/ROCK pathway is dysregulated in SMA, thereby causing a hypophosphorylation of cofilin and a hyperphosphorylation of profilin2^18^. Moreover, cofilin is phosphorylated by LIM kinase (LIMK) which becomes not only activated by RhoA/ROCK, but is also a downstream effector of the other small GTPases Rac1 and Cdc42^40^. Thus, inactivation or dysregulation of one of these pathways may contribute to actin rod formation in SMA. We examined the activation of all three small GTPases by using G-LISA assays (Cytoskeleton), an ELISA-based method to detect the GTP-bound form of the proteins (Fig. 4A–C). Only RhoA showed a significantly increased activation (Fig. 4A), indicating that the RhoA/ROCK axis is the major small GTPase-pathway potentially involved in actin rod formation in SMA. Inhibition of this pathway has already been shown to ameliorate the phenotype in SMA mouse models^41^. Thus, we wanted to assess whether this beneficial effect may be partially mediated by the disassembly of actin rods. ROCK inhibition by 50 µM Y-27632 showed a robust dephosphorylation of cofilin in untransfected cells after 72 h total incubation time (Fig. 4D). PLXND1 staining of scr- and si*Smn*-treated cells with or without inhibitor revealed a significant reduction of the cell number with rods under SMN depletion (Fig. 4E,F). Accordingly, reduced actin rod formation due to ROCK inhibition may contribute to alleviated SMA symptoms.

## Discussion

Recently, our group has demonstrated the presence of actin-cofilin rods in cellular and mouse models of SMA^12^. In this study, we wanted to characterize actin rod composition induced by SMN depletion, thereby identifying putative pathways contributing to actin rod generation. Indeed, we determined the neuronal actin-binding protein profilin2 and its upstream kinase ROCK to be involved in actin rod assembly (Figs. 2 and 4). Moreover, LC–MS-based proteomics revealed novel rod-interacting proteins which are enriched in distinct pathways important for cellular homeostasis (Fig. 1).

Minamide et al. previously analyzed rods from epidermoid carcinoma A431 cells^9^. From those seven proteins in rods, we could confirm the presence of six proteins in our list (Supplementary Table 1): gamma-Actin (Actg1), Cofilin (Cfl1, Cfl2), Peroxiredoxin (Prdx1), Annexin A2 (Anxa2), HSP60 (Hspd1), and 14–3–3 (Ywhaq). This argues that main components in actin rods are conserved between very different conditions (motoneuron-like NSC34 cells vs. epidermoid carcinoma cells). However, Bernstein et al. (2006) induced rods in hippocampal neurons by inhibition of ATP synthesis^1^. In our analysis, ATP-binding and ATPase activity are top hits in the list of gene ontology terms (Fig. 1C).

The actin-binding protein profilin has not been investigated in the context of actin rod formation so far. Our results suggest a specific function of the neuronal isoform profilin2, but not profilin1, in the assembly of stable actin rods (Fig. 2). Although both isoforms are highly similar in their tertiary structure, specific characteristics of profilin2 compared to profilin1 could be responsible for these differential effects^42^. Profilins harbor three domains which mediate their binding to actin, proteins with PLP-stretches and phospholipids^34–38^. The main functional difference between the isoforms is considered to be based on their interaction with distinct protein complexes via their PLP-binding domain^43^. Although both isoforms favor the formation of linear, unbranched actin filaments by their interaction with proteins such as formin or Ena/VASP, profilin1 can additionally promote the assembly of branched filaments mediated by its binding to the WAVE2-/Arp2/3-complex^44–47^. Actin rod formation was suggested to be mediated by cofilin-actin subunit assembly at the barbed end of existing actin filaments and their subsequent bundling^9^. Thus, the contribution of profilin2 but not profilin1 in actin rod formation may be based on the preferential bundling of linear instead of branched actin filaments.

Accordingly, we will expect a decrease in actin rod assembly if the PLP- or actin-binding capacity of profilin2 is disturbed. However, overexpression of profilin2 W3A, a mutant deficient in PLP-binding^39^, did not reduce actin rod formation (Fig. 3D). This apparent contradiction may be explained by the finding that profilin2 dimerizes upon binding to VASP to promote actin polymerization^48^. As knock-down of profilin2 reduced endogenous protein levels by only about 50% (Fig. 2A), endogenous and mutant protein could probably form heterodimers to induce actin assembly. The profilin2 mutant deficient in actin-binding, R74E, showed a tendency towards less actin rod formation (Fig. 3D), potentially due to a simultaneous attenuation in PLP-binding^39^. This would impair its interaction with PLP-proteins such as VASP and reduce the polymerization of linear, unbranched actin filaments. Similarly, the profilin2 phospho-mimetic Y78D showed a tendency towards reduced actin rod formation (Fig. 3C). This mutant has a lower affinity to actin, but did not show any effect on PLP-binding. In contrast, enhanced PIP_2_-binding may compete with its function in actin polymerization^49^.

Little is known about mechanisms leading to nuclear actin rod assembly. Transport of actin monomers into the nucleus is mediated by its binding to cofilin^50^. Moreover, nuclear shuttling of cofilin is essential for actin rod formation^51^. In *Dictyostelium discoideum*, the actin-interacting protein 1 (Aip1) and coronin (corA) were shown to contribute to nuclear actin rod formation by potentially providing a sufficient pool of monomeric actin^52^. This function may also pertain to profilin. In line with that, profilin2 mutants R74E and S71D, which do not bind actin^39,49^, showed reduced actin rod assembly (Fig. 3D). However, profilin2 mutant S129D – deficient in actin-binding, but unaffected in PIP_2_- or PLP-binding^49^—did not show a reduction in actin rod formation (Fig. 3D). Thus, the ability to bind actin may be a strong, but non-exclusive contributing factor of profilin2 as a regulator of actin rod formation.

Profilin2 knock-down significantly reduced the number of SMN-depleted cells with actin rods indicating a role of profilin2 in actin rod assembly under SMA conditions (Fig. 2E). Although there was no regulation of profilin2 levels in a SMA model in NSC34 cells, profilin2 is hyperphosphorylated in SMA cellular and mouse models^18^. We tested several single-site phospho-mutants of profilin2 for their potential to influence actin rod formation (Fig. 3) and identified serine residue 137 of profilin2 as a putative phospho-site enhancing the capacity of the protein to induce actin rod formation (Fig. 3D,E). Interestingly, the analogous phospho-site of profilin1 is targeted by the kinase ROCK1^33^.

Dysregulation of the RhoA/ROCK axis is hypothesized to be the cause for profilin2 hyperphosphorylation and cofilin hypophosphorylation in SMA^18^. Inhibition of this pathway significantly reduced actin rod formation in SMN-depleted cells (Fig. 4F). ROCK inhibition causes a dephosphorylation of its downstream targets such as cofilin^41,53^. As dephosphorylated cofilin is required for actin rod formation^7,8^, we would have expected even enhanced rod assembly in inhibitor-treated compared to control cells. However, ROCK inhibition induced less actin rod formation. Therefore, this effect is putatively mediated by the dephosphorylation of other downstream targets such as profilin2 at S137. Interestingly, inhibition of this pathway ameliorates SMA pathogenesis in mice^41,54^. This beneficial effect may be partly mediated by a reduction in actin rod formation in motoneurons. However, ROCK inhibition regulates several downstream targets in diverse cell types and their combined action may lead to an improved phenotype in SMA^55^.

How does the formation of actin rods affect motoneuron homeostasis? The assembly of cytoplasmic actin rods may induce synaptic loss by blocking axonal transport physically and by disrupting microtubule integrity^10^. Indeed, impairment in axonal transport of mitochondria and mRNAs was observed in SMA^15,16,56^. Interestingly, dysfunction in mitochondria transport was ameliorated by treatment with N-acetylcysteine, an antioxidant shown to dissolve actin rods^15,57^. Nuclear actin rods perturb the localization of chromatin and the RNA polymerase II affecting gene transcription^58,59^. In line with that, transcriptome abnormalities were detected in SMA motoneurons. However, a general inhibition of transcription has not been reported^60,61^. Moreover, cytoskeletal proteins such as actin and cofilin, but also profilin, tropomyosin, WASP and Arp2/3 were detected to be enriched in the actin rod fraction (Supplementary Table 1) and thus probably unavailable for actin dynamics in other cellular compartments, e.g. the synapse. Accordingly, neuromuscular junctions of SMA mice show a delay in maturation and abnormal synaptic transmission^62,63^. Both are processes relying on functional actin polymerization and depolymerization^64,65^. In addition, we identified other proteins bound to actin rods (Supplementary Table 1). Several of them such as peroxiredoxin1, annexin2 or 14–3–3 were already identified in an earlier study analyzing actin rod composition in ATP-depleted cells^9^. GO ontology of the identified proteins based on their molecular functions revealed their involvement in a number of ATP-consuming processes important for cellular homeostasis (Fig. 1C). Thus, an initial binding of these proteins to actin rods may be beneficial for the stressed cell, but if this condition persists, several essential pathways such as ubiquitination, translation or mitochondria respiration may be perturbed affecting motoneuron integrity. All of these pathways are known to be impaired in SMA^16,66,67^.

In summary, our study supports the importance of the dysregulated RhoA/ROCK pathway in the formation of actin rods in SMA. Thereby, profilin2 is involved in the assembly of these structures which is potentially induced by its phosphorylation on serine residue 137. Based on our finding that several proteins involved in essential cellular functions are bound and potentially sequestered by actin rods, it is important to identify strategies for the disassembly of these structures. This supports the need for combinatorial treatments targeting SMN-dependent and -independent pathways as an increase of the SMN level is not sufficient to dissolve actin rods^12^.

## Material and methods

### Cell culture, siRNAs, plasmids and inhibitors

Motoneuron-like NSC34 cells, a murine hybrid cell line of neuroblastoma and spinal cord cells^20^, were maintained at 37 °C and 5% CO_2_. Cells were cultivated in DMEM with high glucose, GlutaMAX and pyruvate (31966, Thermo Fisher Scientific, Waltham, Masssachusetts, USA) which was supplemented with 5% FCS, 100 U/mL penicillin and 0.1 mg streptomycin. About 24 h after seeding, cells were transfected using Lipofectamine2000 (11668019, Thermo Fisher Scientific, Waltham, Massachusetts, USA) for 24-well and 6-well plates or Metafectene Pro (T040, Biontex, Munich, Germany) for 10 cm dishes according to the manufacturer’s instructions. Shortly, 0.5 µg DNA and/or 30 pmol siRNA diluted in 50 µL OptiMEM (31985062, Thermo Fisher Scientific, Waltham, Massachusetts, USA) were mixed with 3 µL Lipofectamine2000 (2 µL for pAAV constructs) in 50 µL OptiMEM, incubated for about 20 min at RT and added drop-wise on the cells seeded in 24-well plates. The concentrations were upscaled according to the used plate formats, e.g. 70 pmol siRNA with 7 µL Lipofectamine2000 for 6-wells and 420 pmol siRNA with 42 µL Metafectene Pro for 10 cm dishes. Knock-down efficiency for Lipofectamine2000 is about 90% as shown previously^12^. Simultaneously, medium was changed to low serum conditions (1% FCS) in which cells were differentiated for three days. Sequences used for transfection: scrambled siRNA: *AUACGAACGGAACGAACAACA*, si*Pfn1*: *CACCAUGAC UGCCAAGACGCU*, si*Pfn2*: *GAGCAUCACGCCAGUAGAAAU*, si*Smn: CAGAAGUAAAGCA CACAGCAA* and shRNA against *Pfn*2 (*GTCCACGCAGGCACAATTAACTCGAGTTAATTGT GCCTGCGTGGACATTTTT* (Forward)). ROCK inhibition was performed using 50 µM Y-27632 (S1049, Selleck Chemicals, Houston, Texas, USA). The inhibitor was added six hours after transfection and changed every 24 h until lysis or fixation.

### SDS-PAGE and Western blot analysis

If not noted differently, cells were washed with PBS, scraped into RIPA buffer supplemented with protease inhibitor (cOmplete Protease Inhibitor Cocktail, 4693132001, Roche, Basel, Switzerland) and phosphatase inhibitor (PhosSTOP, 4906845001, Roche, Basel, Switzerland) and homogenized by passing six times through a 22G needle. After 30 min incubation on ice, cell debris was separated by centrifugation for 20 min at 16,000×*g* and 4 °C. Protein concentration in the supernatant was determined using the Pierce BCA Protein Assay Kit (23225, Thermo Fisher Scientific, Waltham, Massachusetts, USA). Equal amounts of protein were separated by SDS-PAGE and either stained with Coomassie G250 for LC–MS analysis or blotted for one hour at 120 V on a nitrocellulose membrane (Amersham Hybond ECL Nitrocellulose Membrane, GE Healthcare, Chicago, Illinois, USA). For detection, the following antibodies were used: rabbit α-profilin1 (P7624, 1:1000, Sigma-Aldrich, St. Louis, Missouri, USA), rabbit α-profilin2 (P0101, 1:1000, Sigma-Aldrich, St. Louis, Missouri, USA), rabbit α-cofilin (5175, 1:1000, Cell Signaling Technology, Danvers, Massachusetts, USA), rabbit α-p-cofilin (sc-21867, 1:1000, Santa Cruz Biotechnology, Dallas, Texas, USA) as primary antibodies and HRP-conjugated α-rabbit secondary antibody (A-11034, 1:4000, Molecular Probes, Invitrogen, Carlsbad, California, USA). α-profilin1 and α-profilin2 antibodies detect the carboxy terminal region (amino acids 128–140) of the protein respectively. This region shows a sequence similarity of 62.5% between both isoforms (NCBI sequences: profilin1: NP_005013.1, profilin2: NP_444252.1). Bands were visualized using Immobilon Western Chemiluminescent HRP Substrate (WBKLS0500, Millipore, Burlington, Massachusetts, USA). Densitometric analysis was done by using the software LabImage1D (Kapelan, Leipzig, Germany).

### Actin rod isolation for LC–MS analysis

Actin rods were isolated based on protocols described elsewhere^9,12^. Briefly, 1 × 10^6^ cells were seeded in 10 cm dishes, transfected the next day with siRNA against *Smn* and differentiated for three days. Before harvesting the cells, they were incubated with 10 µM nocodazole for 10 min at 37 °C to prevent microtubule interference. Cells were washed with PBS, scraped into 300 µL lysis buffer (20 mM PIPES pH 6.8, 140 mM NaCl, 1 mM EGTA) and disrupted by passing the cells 40 times through a 27G needle. Cell debris was pelleted by centrifugation for 10 min at 350 × g and 4 °C. Actin rods in the supernatant were separated from monomeric actin by centrifugation on a discontinuous OptiPrep gradient (10%/15% or 10%/40% (v/v), 4 mL each) for 20 min at 4300 rpm and 4 °C. 500 µL fractions were collected and further analyzed on Western blot. Proteins in the actin rod containing fraction were precipitated by adding 4 volumes of ice-cold acetone and incubated for one hour at − 20 °C. After centrifugation, the pellet was dissolved in Lämmli buffer and incubated for five minutes at 95 °C. Proteins were alkylated by adding 1 µL acrylamide (40%) and incubation for 30 min at RT prior to loading on an SDS gel.

### LC–MS-based proteome analyses

Proteins separated by SDS-PAGE were stained with Coomassie Brilliant Blue and each lane was cut into three pieces which were further minced to 1 mm^3^ gel pieces. Further sample processing was done as described^68^. Briefly, gel pieces were destained two times with 200 µL 50% ACN, 50 mM ammonium bicarbonate (ABC) at 37 °C for 30 min and then dehydrated with 100% ACN. Solvent was removed in a vacuum centrifuge and 100 µL 10 ng/µL sequencing grade Trypsin (V5111, Promega, Madison, Wisconsin, USA) in 10% ACN, 40 mM ABC were added. Gels were rehydrated in trypsin solution for 1 h on ice and then covered with 10% ACN, 40 mM ABC. Digestion was performed overnight at 37 °C and was stopped by adding 100 µL of 50% ACN, 0.1% TFA. After incubation at 37 °C for 1 h the solution was transferred into a fresh sample vial. This step was repeated twice and extracts were combined and dried in a vacuum centrifuge. Dried peptide extracts were redissolved in 30 µL 2% ACN, 0.1% TFA with shaking at 800 rpm for 20 min. After centrifugation at 20,000 × g aliquots of 12.5 µL each were stored at − 20 °C. Peptide samples were separated with a nano-flow ultra-high pressure liquid chromatography system (RSLC, Thermo Fisher Scientific, Waltham, Massachusetts, USA) equipped with a trapping column (3 µm C18 particle, 2 cm length, 75 µm ID, Acclaim PepMap, Thermo Fisher Scientific, Waltham, Massachusetts, USA) and a 50 cm long separation column (2 µm C18 particle, 75 µm ID, Acclaim PepMap, Thermo Fisher Scientific, Waltham, Massachusetts, USA). Peptide mixtures were injected, enriched and desalted on the trapping column at a flow rate of 6 µL/min with 0.1% TFA for 5 min. The trapping column was switched online with the separating column and peptides were eluted with a multi-step binary gradient of buffer A (0.1% formic acid) and buffer B (80% ACN, 0.1% formic acid). The RSLC system was coupled online via a Nano Spray Flex Ion Soure II (ES071, Thermo Fisher Scientific, Waltham, Massachusetts, USA) to an LTQ-Orbitrap Velos mass spectrometer that was operated in data-dependent acquisition mode. Overview scans were acquired at a resolution of 60 k in the orbitrap analyzer and the top 10 most intensive ions were selected for CID fragmentation. Fragment ion mass spectra were recorded in the LTQ at normal scan. Active exclusion was activated to 70 s. Raw MS data were processed using MaxQuant^69^ and human entries of Uniprot data bases. Proteins were stated identified by a false discovery rate of 0.01 on protein and peptide level.

### Gene ontology (GO) enrichment

GO enrichment was assessed with the Protein Analysis Through Evolutionary Relationship (PANTHER) overrepresentation test (version 14.1)^21–23^. MS analysis identified 2522 proteins in total. We set the cutoff at an intensity of > 100 keeping 517 proteins for the GO enrichment according to their molecular functions. Whole mouse genome was used as reference and statistics was performed with the Fisher exact t-test.

### Immuncytochemistry

Cells were washed with PBS and fixed with 4% (w/v) PFA at RT for 10 min. After extensive washing, cells were permeabilized and blocked with PBS containing 5% horse serum and 0.3% Triton X-100 for at least 10 min at RT. Primary antibodies in 1% horse serum and 0.3% Triton X-100 in PBS were added for 1 h at RT or overnight at 4 °C. Having washed multiple times with PBS, Alexa-coupled secondary antibodies (1:500, Invitrogen, Carlsbad, California, USA) in PBS with 1% horse serum were added for 1 h at RT. Counterstaining of the nucleus was performed with DAPI in PBS for 2 min at RT, before mounting the cover slips in Prolong Gold (Life Technologies, Carlsbad, California, USA). Primary antibodies were: goat α-plexinD1 (ab28762, 1:200, Abcam, Cambridge, UK), rabbit α-profilin1 (P7624, 1:1000, Sigma-Aldrich, St. Louis, Missouri, USA) and rabbit α-profilin2 (P0101, 1:1000, Sigma-Aldrich, St. Louis, Missouri, USA),

### Microscopy and evaluation of images

The percentage of cells with actin rods was determined by staining with goat α-plexinD1 antibody (ab28762, 1:200, Abcam, Cambridge, UK). Epifluorescence images were taken with the Olympus BX60 upright fluorescence microscope equipped with an Olympus XM10 color view camera and Olympus Cell Sense software. Exposure time was kept equal for each channel within an experiment and images were taken randomly. Cell counting was performed manually with blinded samples using ImageJ software.

Confocal images were taken with a Leica DM IRB microscope equipped with a TCS SP2 AOBS scan head, an oil immersion objective HCX PL APO BL (63 ×, numeric aperture 1.4) and a Leica acquisition software. Co-localization in the indicated ROIs were analyzed using the Intensity Correlation Analysis plugin for ImageJ to determine the product of the differences of the mean (PDM) values.

### In silico analysis

Putative phospho-sites of human profilin2a (NCBI reference sequence: NP_444252.1) were determined by using the NetPhos 3.1 program^31,32^. 9 putative phospho-sites were chosen for further analysis.

### Molecular cloning

Human profilin2a cDNA in pCIneo was used to generate profilin2 phospho-mimetics and phospho-mutants. Overlapping primers were designed to exchange single serine, tyrosine and threonine residues for aspartic acid or alanine, respectively, by PCR. These cDNAs were cloned into pIRES2-EGFP by PCR using primers with an added restriction site for *BamH*I and subsequent restriction digestion with *EcoR*I and *BamH*I. Next, PFN2-IRES-EGFP was molecularly cloned into the bicistronic vector pAAV_H1_Syn-EGFP by PCR using primers with added restriction sites for *Age*I and *BsrG*I. Restriction digestion with these two enzymes removed the original EGFP from the plasmid. shRNA against *Pfn2* was cloned behind the H1 promoter by designing the shRNA with restriction sites for BglII and HindIII with the GPP Web Portal of the Broad Institute.

### Small GTPase assays

Cells were treated with scr siRNA or si*Smn* and the activity of small G-proteins (Rac1, RhoA and Cdc42) was measured by utilizing G-LISA Activation Assays (BK135, Cytoskeleton, Denver, Colorado, USA). The assays were performed according to the manufacturer’s instructions. Shortly, cell lysate was added to wells linked with the binding domain of an effector molecule of the small GTPases. The active protein was bound to the well bottom and was subsequently detected via specific small G-protein antibodies. An HRP-linked secondary antibody allowed colorimetric determination of the degree of activation.

### Statistics

The GraphPad PRISM software (GraphPad Software, San Diego, California, USA) was used for all statistical analyzes.

## Supplementary Information

Supplementary Information.

## Abbreviations

SMA: Spinal muscular atrophy
SMN: Survival of motoneuron
PLXND1: PlexinD1
PFN1/2: Profilin1/2
ROCK: RhoA-associated coiled-coil kinase
LIMK: LIM kinase

## References

[1] Bernstein BW, Chen H, Boyle JA, Bamburg JR (2006). Formation of actin-ADF/cofilin rods transiently retards decline of mitochondrial potential and ATP in stressed neurons. Am. J. Physiol. Cell. Physiol..

[2] Bershadsky AD, Gelfand VI, Svitkina TM, Tint IS (1980). Destruction of microfilament bundles in mouse embryo fibroblasts treated with inhibitors of energy metabolism. Exp. Cell. Res..

[3] Ohta Y, Nishida E, Sakai H, Miyamoto E (1989). Dephosphorylation of cofilin accompanies heat shock-induced nuclear accumulation of cofilin. J. Biol. Chem..

[4] Nishida E (1987). Cofilin is a component of intranuclear and cytoplasmic actin rods induced in cultured cells. Proc. Natl. Acad. Sci. USA.

[5] Dedova IV, Nikolaeva OP, Mikhailova VV, dos Remedios CG, Levitsky DI (2004). Two opposite effects of cofilin on the thermal unfolding of F-actin: A differential scanning calorimetric study. Biophys. Chem..

[6] Pfannstiel J (2001). Human cofilin forms oligomers exhibiting actin bundling activity. J. Biol. Chem..

[7] Bernstein BW, Shaw AE, Minamide LS, Pak CW, Bamburg JR (2012). Incorporation of cofilin into rods depends on disulfide intermolecular bonds: Implications for actin regulation and neurodegenerative disease. J. Neurosci..

[8] Minamide LS, Striegl AM, Boyle JA, Meberg PJ, Bamburg JR (2000). Neurodegenerative stimuli induce persistent ADF/cofilin-actin rods that disrupt distal neurite function. Nat. Cell. Biol..

[9] Minamide LS (2010). Isolation and characterization of cytoplasmic cofilin-actin rods. J. Biol. Chem..

[10] Cichon J (2012). Cofilin aggregation blocks intracellular trafficking and induces synaptic loss in hippocampal neurons. J. Biol. Chem..

[11] Munsie L (2011). Mutant huntingtin causes defective actin remodeling during stress: Defining a new role for transglutaminase 2 in neurodegenerative disease. Hum. Mol. Genet..

[12] Rademacher S (2017). Metalloprotease-mediated cleavage of PlexinD1 and its sequestration to actin rods in the motoneuron disease spinal muscular atrophy (SMA). Hum. Mol. Genet..

[13] Lefebvre S (1995). Identification and characterization of a spinal muscular atrophy-determining gene. Cell.

[14] D'Amico A, Mercuri E, Tiziano FD, Bertini E (2011). Spinal muscular atrophy. Orphanet. J. Rare Dis..

[15] Xu CC, Denton KR, Wang ZB, Zhang X, Li XJ (2016). Abnormal mitochondrial transport and morphology as early pathological changes in human models of spinal muscular atrophy. Dis. Model. Mech..

[16] Miller N, Shi H, Zelikovich AS, Ma YC (2016). Motor neuron mitochondrial dysfunction in spinal muscular atrophy. Hum. Mol. Genet..

[17] Acsadi G (2009). Mitochondrial dysfunction in a neural cell model of spinal muscular atrophy. J. Neurosci. Res..

[18] Nölle A (2011). The spinal muscular atrophy disease protein SMN is linked to the Rho-kinase pathway via profilin. Hum. Mol. Genet..

[19] Luchino J (2013). Semaphorin 3E suppresses tumor cell death triggered by the plexin D1 dependence receptor in metastatic breast cancers. Cancer Cell.

[20] Cashman NR (1992). Neuroblastoma x spinal cord (NSC) hybrid cell lines resemble developing motor neurons. Dev. Dyn..

[21] Thomas PD (2003). PANTHER: A library of protein families and subfamilies indexed by function. Genome Res..

[22] Mi H, Muruganujan A, Ebert D, Huang X, Thomas PD (2018). PANTHER version 14: More genomes, a new PANTHER GO-slim and improvements in enrichment analysis tools. Nucleic Acids Res..

[23] Mi H, Muruganujan A, Thomas PD (2013). PANTHER in 2013: Modeling the evolution of gene function, and other gene attributes, in the context of phylogenetic trees. Nucleic Acids Res..

[24] Haas AL, Rose IA (1982). The mechanism of ubiquitin activating enzyme: A kinetic and equilibrium analysis. J. Biol. Chem..

[25] Jewett MC, Miller ML, Chen Y, Swartz JR (2009). Continued protein synthesis at low [ATP] and [GTP] enables cell adaptation during energy limitation. J. Bacteriol..

[26] Mayer MP (2010). Gymnastics of molecular chaperones. Mol. Cell.

[27] Ardito F, Giuliani M, Perrone D, Troiano G, LoMuzio L (2017). The crucial role of protein phosphorylation in cell signaling and its use as targeted therapy (Review). Int. J. Mol. Med..

[28] Cao LG, Babcock GG, Rubenstein PA, Wang YL (1992). Effects of profilin and profilactin on actin structure and function in living cells. J. Cell. Biol..

[29] Giesemann T (1999). A role for polyproline motifs in the spinal muscular atrophy protein SMN: Profilins bind to and colocalize with smn in nuclear gems. J. Biol. Chem..

[30] Sharma A (2005). A role for complexes of survival of motor neurons (SMN) protein with gemins and profilin in neurite-like cytoplasmic extensions of cultured nerve cells. Exp. Cell. Res..

[31] Blom N, Gammeltoft S, Brunak S (1999). Sequence- and Structure-based prediction of eukaryotic protein phosphorylation sites. J. Mol. Biol..

[32] Blom N, Sicheritz-Ponten T, Gupta R, Gammeltoft S, Brunak S (2004). Prediction of post-translational glycosylation and phosphorylation of proteins from the amino acid sequence. Proteomics.

[33] Shao J, Welch WJ, Diprospero NA, Diamond MI (2008). Phosphorylation of profilin by ROCK1 regulates polyglutamine aggregation. Mol. Cell. Biol..

[34] Carlsson L (1976). Crystallization of a non-muscle actin. J. Mol. Biol..

[35] Carlsson L, Nyström LE, Sundkvist I, Markey F, Lindberg U (1977). Actin polymerizability is influenced by profilin, a low molecular weight protein in non-muscle cells. J. Mol. Biol..

[36] Lassing I, Lindberg U (1985). Specific interaction between phosphatidylinositol 4,5-bisphosphate and profilactin. Nature.

[37] Tanaka M, Shibata H (1985). Poly(L-proline)-binding proteins from chick embryos are a profilin and a profilactin. Eur. J. Biochem..

[38] Metzler WJ, Bell AJ, Ernst E, Lavoie TB, Mueller L (1994). Identification of the poly-L-proline-binding site on human profilin. J. Biol. Chem..

[39] Lambrechts A, Jonckheere V, Dewitte D, Vandekerckhove J, Ampe C (2002). Mutational analysis of human profilin I reveals a second PI(4,5)-P2 binding site neighbouring the poly(L-proline) binding site. BMC Biochem..

[40] Luo L (2002). Actin cytoskeleton regulation in neuronal morphogenesis and structural plasticity. Annu. Rev. Cell Dev. Biol..

[41] Bowerman M, Beauvais A, Anderson CL, Kothary R (2010). Rho-kinase inactivation prolongs survival of an intermediate SMA mouse model. Hum. Mol. Genet..

[42] Nodelman IM, Bowman GD, Lindberg U, Schutt CE (1999). X-ray structure determination of human profilin II: A comparative structural analysis of human profilins11Edited by R. Huber. J. Mol. Biol..

[43] Witke W (1998). In mouse brain profilin I and profilin II associate with regulators of the endocytic pathway and actin assembly. EMBO J..

[44] Miki H, Suetsugu S, Takenawa T (1998). WAVE, a novel WASP-family protein involved in actin reorganization induced by Rac. Embo J..

[45] Yang C (2000). Profilin enhances Cdc42-induced nucleation of actin polymerization. J. Cell Biol..

[46] Mouneimne G (2012). Differential remodeling of actin cytoskeleton architecture by profilin isoforms leads to distinct effects on cell migration and invasion. Cancer Cell.

[47] Suarez C (2015). Profilin regulates F-actin network homeostasis by favoring formin over Arp2/3 complex. Dev. Cell.

[48] Jonckheere V, Lambrechts A, Vandekerckhove J, Ampe C (1999). Dimerization of profilin II upon binding the (GP5)3 peptide from VASP overcomes the inhibition of actin nucleation by profilin II and thymosin β4. FEBS Lett..

[49] Walter LM (2019). Profilin2a-phosphorylation as a regulatory mechanism for actin dynamics. FASEB.

[50] Dopie J, Skarp KP, Rajakyla EK, Tanhuanpaa K, Vartiainen MK (2012). Active maintenance of nuclear actin by importin 9 supports transcription. Proc. Natl. Acad. Sci. USA.

[51] Munsie LN, Desmond CR, Truant R (2012). Cofilin nuclear–cytoplasmic shuttling affects cofilin–actin rod formation during stress. J. Cell Sci..

[52] Ishikawa-Ankerhold HC, Daszkiewicz W, Schleicher M, Müller-Taubenberger A (2017). Actin-interacting protein 1 contributes to intranuclear rod assembly in dictyostelium discoideum. Sci. Rep..

[53] Maekawa M (1999). Signaling from rho to the actin cytoskeleton through protein kinases ROCK and LIM-kinase. Science.

[54] Bowerman M, Murray L, Boyer J, Anderson CL, Kothary R (2012). Fasudil improves survival and promotes skeletal muscle development in a mouse model of spinal muscular atrophy. BMC Med..

[55] Coque E, Raoul C, Bowerman M (2014). ROCK inhibition as a therapy for spinal muscular atrophy: Understanding the repercussions on multiple cellular targets. Front. Neurosci..

[56] Fallini C, Bassell GJ, Rossoll W (2012). Spinal muscular atrophy: The role of SMN in axonal mRNA regulation. Brain Res..

[57] Tam S-W (2019). Endothelin type B receptor promotes cofilin rod formation and dendritic loss in neurons by inducing oxidative stress and cofilin activation. J. Biol. Chem..

[58] Serebryannyy LA (2016). Persistent nuclear actin filaments inhibit transcription by RNA polymerase II. J. Cell Sci..

[59] Serebryannyy LA, Yuen M, Parilla M, Cooper ST, de Lanerolle P (2016). The effects of disease models of nuclear actin polymerization on the nucleus. Front. Physiol..

[60] Zhang Z (2008). SMN deficiency causes tissue-specific perturbations in the repertoire of snRNAs and widespread defects in splicing. Cell.

[61] Murray LM (2010). Pre-symptomatic development of lower motor neuron connectivity in a mouse model of severe spinal muscular atrophy. Hum. Mol. Genet..

[62] Kariya S (2008). Reduced SMN protein impairs maturation of the neuromuscular junctions in mouse models of spinal muscular atrophy. Hum. Mol. Genet..

[63] Kong L (2009). Impaired synaptic vesicle release and immaturity of neuromuscular junctions in spinal muscular atrophy mice. J. Neurosci..

[64] Zhang W, Benson DL (2001). Stages of synapse development defined by dependence on F-actin. J. Neurosci..

[65] Dillon C, Goda Y (2005). THE actin cytoskeleton: Integrating form and function at the synapse. Annu. Rev. Neurosci..

[66] Wishart TM (2014). Dysregulation of ubiquitin homeostasis and beta-catenin signaling promote spinal muscular atrophy. J. Clin. Invest..

[67] Bernabò P (2017). In vivo translatome profiling in spinal muscular atrophy reveals a role for SMN protein in ribosome biology. Cell Rep..

[68] Jochim N, Gerhard R, Just I, Pich A (2011). Impact of clostridial glucosylating toxins on the proteome of colonic cells determined by isotope-coded protein labeling and LC-MALDI. Proteome Sci..

[69] Cox J, Mann M (2008). MaxQuant enables high peptide identification rates, individualized p.p.b.-range mass accuracies and proteome-wide protein quantification. Nat. Biotechnol..

