# Supplementary material for: Profilin2 regulates actin rod assembly in neuronal cells
Source: Sci Rep. 2021 May 13;11:10287. doi: 10.1038/s41598-021-89397-9 (PMC8119500; doi:10.1038/s41598-021-89397-9)

**Profilin2 regulates actin rod assembly in neuronal cells**

## Supplementary material

Lisa Marie Walter^1,4^, Sebastian Rademacher^1,2,4^_,_ Andreas Pich^3^ & Peter Claus^1,4,*^

^1^ Institute of Neuroanatomy and Cell Biology, Hannover Medical School, Hannover, Germany.

^2^ present address: Institute of Biochemistry, Charité-Universitätsmedizin Berlin, Germany.

^3^ Institute of Toxicology and Core Unit Proteomics, Hannover Medical School, Hannover, Germany.

^4^ Center for Systems Neuroscience, Hannover, Germany.

^*^email: claus.peter@mh-hannover.de

# Supplementary tables

Supplementary table 1: List of proteins identified to be bound to actin rods by LC-MS.

The table was simplified by using the names of the respective protein-coding genes. Different histone variants with similar molecular weights were not itemized. Proteins with a total intensity below 100 were not considered in further analyzes.

| **Gene name** | **Molecular weight**  **(kDa)** | **Total intensity** | **Gene name** | **Molecular weight**  **(kDa)** | **Total intensity** |
| --- | --- | --- | --- | --- | --- |
| Actg1 | 41.79 | 235.33 | Hnrnpa1 | 38.83 | 188.39 |
| Eef1a1 | 50.11 | 233.93 | Ddx39b | 49.04 | 188.33 |
| Tuba1b/4a/8 | 50.15 | 229.70 | Eef2 | 95.31 | 188.13 |
| Hspa8 | 70.87 | 222.87 | Hnrnpk | 48.56 | 188.13 |
| Rps27a | 17.95 | 222.44 | Slc25a3 | 39.74 | 188.08 |
| Tubb5 | 49.67 | 221.29 | Hist1h4a | 11.37 | 187.34 |
| Gapdh | 35.81 | 213.71 | Eef1g | 50.06 | 186.77 |
| Hsp90ab1 | 83.28 | 211.77 | Hnrnpa2b1 | 37.40 | 186.74 |
| Npm1 | 28.39 | 211.12 | Cct8 | 53.08 | 186.64 |
| Eno1 | 47.14 | 210.39 | Eif4b | 68.84 | 186.48 |
| Aldoa;Aldoart1 | 39.36 | 206.53 | Nudc | 38.36 | 186.16 |
| Ncl | 76.72 | 202.49 | Myl6 | 16.93 | 186.01 |
| Pkm | 57.84 | 202.44 | Rpl4 | 47.15 | 185.80 |
| Atp5b | 56.30 | 201.97 | Vdac3 | 30.70 | 185.00 |
| Ldha | 36.50 | 201.22 | Cfl1;Cfl2 | 18.56 | 184.76 |
| Hspa9 | 73.46 | 200.95 | Nap1l1 | 48.54 | 184.32 |
| Krt76 | 62.84 | 200.53 | Srsf3;Gm12355 | 14.20 | 184.23 |
| Pgk1 | 44.55 | 199.49 | Arhgdia | 23.41 | 184.06 |
| Hspd1 | 60.96 | 199.29 | Hist1h2 | 14.89 | 183.78 |
| Ppia | 17.97 | 198.67 | Rpl3 | 46.11 | 183.60 |
| Gdi2 | 50.54 | 198.01 | Pcbp1 | 37.50 | 183.52 |
| Cycs | 11.61 | 197.67 | Atp5a1 | 59.75 | 183.11 |
| Slc25a4 | 32.90 | 197.00 | Lgals1 | 14.87 | 183.05 |
| Ran | 24.42 | 196.97 | Cyb5r3 | 34.93 | 183.01 |
| Ywhae | 29.17 | 196.85 | Ahcy | 47.69 | 182.82 |
| Vdac1 | 30.76 | 195.93 | Ppp2r1a | 65.32 | 182.73 |
| Dpysl3 | 73.88 | 195.77 | Vim | 53.69 | 182.43 |
| Hnrnpab | 33.82 | 194.74 | Rpl7a | 29.98 | 182.31 |
| Aco2 | 85.46 | 194.52 | Snrpa | 31.84 | 182.28 |
| Pabpc1 | 70.67 | 194.24 | Mdh2 | 35.61 | 182.14 |
| Cct3 | 60.63 | 193.90 | Rps3 | 26.67 | 182.09 |
| Srsf2 | 25.48 | 193.72 | Rpl22 | 14.76 | 181.94 |
| Vdac2 | 31.73 | 193.19 | Rangap1 | 63.53 | 181.56 |
| Srsf7 | 17.89 | 192.77 | Srm | 34.00 | 181.54 |
| Hnrnpm | 73.74 | 192.70 | Rps6 | 28.68 | 181.13 |
| Aars | 106.91 | 192.34 | Iap | 62.75 | 180.74 |
| Ywhaz | 27.77 | 191.99 | Rpl7 | 31.42 | 180.66 |
| Tufm | 49.54 | 191.25 | Nars | 64.28 | 180.47 |
| Actbl2 | 42.00 | 190.86 | Eif3l | 66.61 | 180.43 |
| Eif4a1 | 46.15 | 190.60 | Fscn1 | 54.51 | 180.41 |
| Lmna | 74.24 | 189.88 | Prkar1a | 43.19 | 179.98 |
| Cs | 51.74 | 188.77 | Rps2 | 31.23 | 179.84 |
| Rpl6 | 33.51 | 188.57 | Acot7 | 42.83 | 179.62 |
| Asns | 64.28 | 179.60 | Tcea1 | 33.88 | 164.78 |
| Mdh1 | 36.51 | 179.16 | Eif5a | 16.83 | 164.30 |
| Pa2g4 | 43.70 | 179.14 | Rars | 75.67 | 164.27 |
| Tars | 83.36 | 178.09 | Rplp0 | 34.22 | 164.26 |
| Hnrnpa0 | 30.53 | 178.04 | Tubb4b;Tubb4a | 49.83 | 164.17 |
| Rps9 | 22.59 | 177.91 | Sub1 | 14.43 | 163.80 |
| Nsfl1c | 40.71 | 177.67 | Cct7 | 55.06 | 163.44 |
| Stip1 | 62.58 | 177.56 | Eif2s2 | 38.09 | 162.21 |
| Rdx | 68.54 | 177.07 | Fus | 52.60 | 161.06 |
| Tomm70a | 67.59 | 176.86 | Eef1d | 27.22 | 160.62 |
| Rpl18 | 21.64 | 176.71 | Timm44 | 51.09 | 160.41 |
| Rps8 | 24.21 | 176.70 | Akr1b1 | 35.73 | 160.39 |
| Vgf | 68.23 | 176.55 | Psmc2 | 48.65 | 159.96 |
| Ranbp1 | 23.60 | 176.18 | Prph | 54.30 | 159.91 |
| Shmt2 | 55.76 | 175.43 | Hnrnpa3 | 34.48 | 159.71 |
| Pdlim5 | 63.30 | 175.42 | Stxbp1 | 67.57 | 159.12 |
| Gars | 81.88 | 174.27 | Tpm3 | 29.02 | 158.93 |
| Hnrnpf | 45.73 | 173.67 | Ppa1 | 32.67 | 158.49 |
| Dpysl2 | 62.28 | 173.52 | Psmc3 | 49.55 | 158.45 |
| Ssb | 47.76 | 172.84 | Tmpo | 75.17 | 158.37 |
| Idh3a | 39.64 | 172.82 | Pebp1 | 20.83 | 158.18 |
| Hmgb1 | 24.23 | 172.55 | Phgdh | 56.59 | 158.11 |
| Srsf1 | 28.33 | 171.55 | Tuba1a;Tuba3a | 50.14 | 158.02 |
| Rpsa | 32.84 | 170.93 | Hspe1 | 10.96 | 157.99 |
| Hspa5 | 72.42 | 170.92 | Atp5c1 | 30.26 | 157.79 |
| Yars | 63.00 | 170.89 | Pcbp2 | 38.22 | 157.62 |
| Nucks1 | 26.31 | 170.59 | Pcna | 28.79 | 157.48 |
| Bcat1 | 43.52 | 170.56 | Rpl27 | 15.80 | 157.44 |
| Hnrnph1 | 51.22 | 170.25 | Dlat | 67.94 | 157.38 |
| Got2 | 47.41 | 170.24 | Uchl1 | 24.84 | 157.36 |
| Ywhag | 28.30 | 170.17 | Rps23 | 15.81 | 157.23 |
| Naca | 23.38 | 170.14 | Tfg | 43.02 | 157.09 |
| Ywhaq | 32.22 | 169.95 | Cbx5 | 22.19 | 156.89 |
| Cttn | 61.25 | 169.78 | Prdx1 | 22.18 | 156.72 |
| Tcp1 | 60.45 | 169.21 | Rbm14 | 69.45 | 156.67 |
| Prep | 80.75 | 168.56 | Aldh9a1 | 53.51 | 156.37 |
| Cct6a | 58.00 | 168.53 | Glod4 | 31.21 | 155.94 |
| Rbmxl1;Rbmx | 42.16 | 168.50 | Hist1h1c | 21.27 | 155.77 |
| St13 | 41.66 | 167.66 | Cct4 | 54.86 | 155.58 |
| Rtn4 | 38.40 | 167.16 | Hsp90aa1 | 84.79 | 155.40 |
| Serbp1 | 22.70 | 167.15 | Cotl1 | 15.94 | 155.25 |
| Sept9 | 64.77 | 167.12 | Fxr1 | 72.81 | 155.20 |
| Hist1h2a | 13.66 | 167.04 | Rpl29 | 16.85 | 155.14 |
| Srsf5 | 30.98 | 166.53 | Psmc6 | 44.17 | 155.11 |
| Anxa6 | 75.88 | 166.52 | Nme2;Nme1 | 30.20 | 155.10 |
| Tubb3 | 50.42 | 165.94 | Cct2 | 57.48 | 155.01 |
| Atic | 64.22 | 165.83 | Trap1 | 80.21 | 154.82 |
| Khsrp | 76.78 | 165.69 | Rps26 | 13.02 | 154.58 |
| Rpl5 | 34.40 | 165.60 | Anxa5 | 35.75 | 154.40 |
| Hspa4 | 94.21 | 165.00 | Eif3g | 35.64 | 153.94 |
| Psip1 | 59.70 | 164.81 | Ass1 | 46.58 | 153.86 |
| Anxa2 | 38.68 | 153.83 | Fubp1 | 67.44 | 140.06 |
| Mtpn | 12.86 | 153.54 | Nans | 40.02 | 139.59 |
| Psmd13 | 42.81 | 153.15 | Ola1 | 44.73 | 139.34 |
| Eif2s1 | 36.11 | 153.01 | Tardbp | 44.55 | 138.81 |
| Psmd3 | 60.72 | 152.95 | Ddx3x;Ddx3y;  D1Pas1 | 73.10 | 138.30 |
| Asl | 51.74 | 152.94 | Rpl10a | 24.83 | 137.75 |
| Uqcrc2 | 48.23 | 152.93 | Vat1 | 43.10 | 137.53 |
| Calr | 47.99 | 152.74 | Hsdl1 | 36.87 | 137.49 |
| Thop1 | 78.03 | 152.60 | Rps25 | 13.74 | 137.41 |
| Aimp2 | 35.38 | 152.34 | Asna1 | 38.82 | 137.30 |
| Fdps | 95.22 | 152.18 | Sae1 | 38.62 | 137.26 |
| Hnrnpc | 32.22 | 150.92 | Anp32b | 31.08 | 137.25 |
| Hist1h1e | 21.98 | 150.17 | Pfkp | 85.55 | 137.24 |
| Gnb2 | 37.33 | 150.02 | Ahsa1 | 38.12 | 137.11 |
| Snrnp35 | 29.29 | 149.67 | Calu | 37.06 | 136.73 |
| Rps15a | 14.84 | 149.26 | Uqcrc1 | 52.85 | 136.66 |
| Tkt | 67.63 | 149.12 | Gmps | 76.72 | 136.22 |
| Marcks | 29.66 | 148.83 | Pacsin2 | 55.83 | 136.17 |
| Eef1b;Eef1b2 | 24.69 | 148.47 | Capzb | 30.63 | 136.07 |
| Krt28 | 50.35 | 148.17 | Oat | 48.35 | 135.93 |
| Taldo1 | 37.39 | 148.06 | Pdia4 | 72.37 | 135.91 |
| Dnajc8 | 27.49 | 147.34 | Syncrip | 58.75 | 135.84 |
| Hist1h3 | 13.32 | 147.33 | Rps3a | 29.89 | 135.70 |
| Rpl13 | 24.31 | 147.14 | Aimp1 | 35.17 | 135.64 |
| Ube2n | 17.14 | 146.90 | Fabp5 | 15.14 | 135.23 |
| Psmc5 | 45.63 | 146.23 | Rpl8 | 28.02 | 135.06 |
| Rpa1 | 71.41 | 146.15 | Park7 | 18.47 | 135.02 |
| Cars | 85.55 | 145.80 | Set | 24.92 | 134.82 |
| Lrpap1 | 42.22 | 145.59 | Pgd | 53.25 | 134.75 |
| Immt | 82.93 | 144.28 | Rpl34 | 13.29 | 134.61 |
| Ddx5 | 69.27 | 144.03 | Glrx3 | 37.78 | 134.47 |
| Rplp2 | 11.65 | 143.94 | Etfa | 35.01 | 134.37 |
| Eef1a2 | 50.45 | 143.43 | Rps12 | 14.52 | 134.24 |
| Rpl23 | 14.87 | 143.42 | Prdx2 | 16.07 | 134.01 |
| Ybx1 | 35.73 | 143.20 | Por | 77.04 | 133.70 |
| Tomm34 | 34.28 | 142.53 | Rpn1 | 68.53 | 133.70 |
| Psat1 | 40.47 | 142.17 | Psma6 | 27.37 | 133.67 |
| Abce1 | 67.31 | 142.09 | Ppa2 | 37.99 | 133.63 |
| Sept2 | 41.53 | 142.09 | Rpl13a | 23.46 | 133.58 |
| Luc7l2 | 38.61 | 141.94 | Nsf | 82.61 | 133.18 |
| Slc25a5 | 32.93 | 141.94 | Blmh | 52.51 | 133.12 |
| Gnb2l1 | 35.08 | 141.66 | Hmgb2 | 24.16 | 133.08 |
| Vars | 141.41 | 141.30 | 4930550L24Rik | 35.04 | 132.98 |
| 2210016F16Rik | 38.62 | 141.09 | Stx1a | 29.49 | 132.93 |
| Psmd11 | 47.44 | 140.97 | Elavl1 | 36.17 | 132.73 |
| Txn | 11.68 | 140.91 | Gap43 | 23.63 | 132.47 |
| Hdgf | 26.27 | 140.87 | Phb | 29.82 | 132.24 |
| Ywhah | 28.21 | 140.68 | Tsn | 26.20 | 132.14 |
| Fh | 50.05 | 140.47 | Msn | 67.77 | 132.12 |
| Cd47 | 33.10 | 140.44 | Wdr1 | 66.41 | 131.99 |
| Snrnp70 | 51.99 | 131.93 | Cbwd1 | 43.77 | 122.78 |
| Psmd7 | 36.54 | 131.86 | Cox5b | 13.85 | 122.38 |
| Rps19 | 16.09 | 131.82 | Cdc37 | 44.59 | 122.29 |
| Sars | 21.26 | 131.79 | Map1b | 270.25 | 122.23 |
| Phb2 | 33.30 | 131.64 | Mydgf | 17.98 | 121.35 |
| Carkd | 35.17 | 131.48 | Hist1h1d | 22.10 | 121.30 |
| Raly | 31.69 | 131.45 | Rps16 | 16.45 | 121.21 |
| Vcp | 89.32 | 131.36 | Psma1 | 29.55 | 121.05 |
| Plod3 | 84.92 | 131.19 | Rps13 | 17.22 | 120.97 |
| Drg2 | 40.72 | 131.18 | Hnrnpd | 32.75 | 120.91 |
| Lrrc59 | 34.88 | 131.08 | Cops4 | 46.28 | 120.83 |
| Gtf2i | 103.08 | 130.50 | Tpi1 | 32.19 | 120.79 |
| Canx | 67.28 | 130.15 | Rtn3 | 25.43 | 120.74 |
| Nop56 | 64.46 | 130.14 | Rps24 | 15.07 | 120.45 |
| Cyp20a1 | 52.15 | 130.11 | Tra2b | 21.94 | 120.17 |
| Nutf2 | 14.48 | 130.02 | Cox4i1 | 19.53 | 119.75 |
| Rpl23a | 17.70 | 130.01 | Pdxp | 31.51 | 119.74 |
| Basp1 | 22.09 | 129.93 | Rpl14 | 23.56 | 119.67 |
| Actr3 | 47.36 | 129.93 | Mthfd2 | 37.86 | 119.55 |
| Ppm1a | 42.43 | 129.80 | Cdk1 | 34.11 | 119.43 |
| Ddah1 | 31.38 | 129.26 | Ppp2cb;Ppp2ca | 35.58 | 119.37 |
| Ptbp1 | 52.63 | 129.22 | Pcbp3 | 39.17 | 119.37 |
| Ndc80 | 73.96 | 129.07 | Klc1 | 61.63 | 119.02 |
| Renbp | 47.98 | 129.04 | Sgta | 34.32 | 119.00 |
| Cbr3 | 30.95 | 128.84 | Stub1 | 34.91 | 118.92 |
| Gstm2 | 25.72 | 128.50 | Dnm1l | 82.66 | 118.87 |
| Clpp | 29.80 | 128.49 | Ppp1ca | 37.54 | 118.59 |
| Cpsf6 | 59.31 | 128.46 | Psmd6 | 45.54 | 118.52 |
| Sh3gl1 | 41.52 | 127.74 | Rpl31 | 14.46 | 118.35 |
| Adrm1 | 42.15 | 127.62 | Idh2 | 50.91 | 118.24 |
| Rbbp7 | 46.91 | 127.46 | Tbcb | 27.39 | 118.23 |
| Eprs | 170.08 | 127.16 | Eif2s3 | 51.07 | 118.20 |
| Scrn1 | 46.33 | 126.61 | S100a6 | 10.05 | 118.14 |
| Khdrbs1 | 48.37 | 126.40 | Bag3 | 61.86 | 118.08 |
| Timm50 | 39.78 | 126.30 | Aacs | 75.20 | 118.07 |
| Psph | 25.10 | 126.20 | Uba1 | 117.81 | 117.89 |
| Rpl28 | 15.73 | 125.80 | Sec61a1 | 52.26 | 117.66 |
| Nono | 54.54 | 125.56 | Hnrnpu | 86.81 | 117.52 |
| Mtco2 | 25.98 | 125.47 | D10Jhu81e | 28.09 | 117.39 |
| Tbca | 12.76 | 125.41 | Vps35 | 91.71 | 117.20 |
| Acat1 | 44.82 | 125.16 | Crmp1 | 74.22 | 117.18 |
| Vat1l | 45.82 | 124.78 | Map2k1 | 43.47 | 116.99 |
| Cct5 | 59.62 | 124.71 | Bzw1 | 51.21 | 116.90 |
| Kars | 67.84 | 124.69 | Trnt1 | 49.90 | 116.79 |
| Gnas | 45.66 | 123.98 | Ctps1 | 66.68 | 116.75 |
| Fbl | 34.31 | 123.89 | Cd63 | 25.77 | 116.72 |
| Tubb2a | 49.91 | 123.85 | Sept7 | 50.68 | 116.70 |
| Psmc4 | 47.41 | 123.74 | Elavl4;Elavl7 | 39.27 | 116.57 |
| Psmd4 | 41.05 | 122.95 | Ddx17 | 72.58 | 116.55 |
| Capza2 | 32.97 | 122.88 | Scamp1 | 32.34 | 116.19 |
| Prkaca | 40.57 | 122.85 | Zyx | 57.03 | 116.06 |
| Prdx4 | 31.05 | 115.97 | Gaa | 106.25 | 110.49 |
| Psme3 | 29.51 | 115.87 | Pdlim7 | 50.12 | 110.18 |
| Heatr3 | 74.31 | 115.77 | Eif3e | 52.22 | 109.92 |
| Pdhb | 38.94 | 115.74 | Atp5o | 23.36 | 109.89 |
| Etf1 | 49.03 | 115.58 | Cisd2 | 15.24 | 109.70 |
| Kifc5b | 73.68 | 115.30 | Cops2 | 51.60 | 109.59 |
| Ruvbl2 | 51.11 | 115.22 | Psmd10 | 25.08 | 109.54 |
| Snrpb2 | 25.32 | 115.20 | Hsph1 | 91.68 | 109.46 |
| Prkcsh | 58.79 | 114.99 | Fhl3 | 31.17 | 109.36 |
| Pfn2 | 9.80 | 114.94 | ? (C2orf47 homolog) | 32.99 | 109.34 |
| Lta4h | 69.05 | 114.75 | Arpc5 | 16.29 | 109.31 |
| Pcmt1 | 29.17 | 114.51 | Akr1b8 | 36.12 | 109.22 |
| Picalm | 70.98 | 114.34 | Myg1 | 42.72 | 109.03 |
| Rhot1 | 72.24 | 114.32 | Xrcc5 | 83.06 | 109.00 |
| Acadl | 47.91 | 114.32 | Tpm4 | 28.47 | 108.94 |
| Tfrc | 85.73 | 114.27 | Pgam | 28.83 | 108.75 |
| Mtdh | 63.85 | 114.04 | Ppp3ca | 57.61 | 108.74 |
| Acat | 41.30 | 114.02 | Tars2 | 72.56 | 108.43 |
| Acp6 | 47.62 | 113.79 | Itpa | 21.90 | 108.31 |
| Hba | 15.11 | 113.30 | Creb1 | 30.96 | 108.19 |
| Ctbp2 | 45.97 | 113.02 | Samhd1 | 72.65 | 107.91 |
| Actr2 | 44.76 | 112.94 | Fmr1 | 66.53 | 107.87 |
| Actl6a | 47.45 | 112.76 | Ttll12 | 74.04 | 107.78 |
| Slc25a1 | 33.93 | 112.72 | Cndp2 | 52.77 | 107.78 |
| Erp44 | 46.85 | 112.59 | Ckap4 | 63.69 | 107.72 |
| Anp32a | 26.86 | 112.55 | Cald1 | 60.45 | 106.72 |
| Rpap3 | 74.10 | 112.53 | Lasp1 | 29.99 | 106.51 |
| Pspc1 | 58.76 | 112.43 | Akr1b10 | 35.85 | 106.50 |
| Rpl19 | 23.25 | 112.38 | Zranb2 | 33.26 | 106.29 |
| Arpc2 | 34.36 | 112.31 | Adsl | 53.13 | 106.28 |
| Crk | 33.81 | 112.19 | Sod1 | 15.94 | 106.03 |
| Suclg2 | 46.84 | 112.18 | Gnaq | 42.16 | 105.85 |
| Rpl37a | 10.28 | 112.01 | Bpnt1 | 33.20 | 105.54 |
| Hnrnpr | 70.89 | 111.99 | Pdha1 | 43.23 | 105.37 |
| Aldh18a1 | 87.05 | 111.61 | Atp1b3 | 31.78 | 105.26 |
| Alyref | 26.94 | 111.53 | Myo16 | 207.66 | 104.95 |
| Map2 | 52.68 | 111.47 | Elmod2 | 34.75 | 104.86 |
| Stmn1 | 17.27 | 111.19 | Surf4 | 30.38 | 104.57 |
| Polb | 38.29 | 111.16 | Rps18 | 17.67 | 104.41 |
| Ezr | 69.41 | 111.10 | Rps7 | 21.88 | 104.40 |
| Tagln2 | 22.40 | 111.02 | Kct2 | 27.29 | 104.32 |
| Hadhb | 51.39 | 110.95 | Ranbp3 | 52.57 | 104.32 |
| Srp68 | 70.57 | 110.94 | Eif3m | 42.52 | 104.19 |
| Arpc1b | 41.50 | 110.90 | Gstp1 | 23.61 | 104.15 |
| Ak1 | 21.54 | 110.87 | Pard6b | 33.27 | 103.99 |
| Fam49b | 36.78 | 110.66 | Ppp1r7 | 41.29 | 103.87 |
| Cfdp1 | 32.92 | 110.63 | Cops3 | 47.83 | 103.76 |
| Ctsd | 44.95 | 110.62 | Myadm | 8.64 | 103.59 |
| Osgep | 19.83 | 110.60 | Rhoa | 21.78 | 103.49 |
| Nme1 | 17.21 | 110.59 | Tpt1 | 19.46 | 103.44 |
| Acaa2 | 41.83 | 110.54 | Xpnpep1 | 70.99 | 103.43 |
| Rps21 | 9.14 | 103.40 | Ndufs1 | 79.78 | 101.26 |
| Pes1 | 68.23 | 102.96 | Qki | 21.43 | 101.06 |
| Bcap29 | 27.96 | 102.92 | Hint1 | 13.78 | 100.89 |
| Prmt1 | 40.52 | 102.89 | Aprt | 19.72 | 100.85 |
| Sh3bgrl | 12.81 | 102.79 | Cdv3 | 24.20 | 100.65 |
| Atp6v1a | 68.33 | 102.57 | Emb | 37.06 | 100.52 |
| Actr1b | 42.28 | 102.38 | Snx2 | 58.47 | 100.44 |
| Sncg | 13.16 | 102.28 | Adss | 50.02 | 100.43 |
| Mettl13 | 78.76 | 102.25 | Prdx6 | 24.83 | 100.34 |
| Dnajc9 | 30.06 | 101.87 | Mat2a | 43.69 | 100.30 |
| Psma7 | 27.86 | 101.72 | Mcm7 | 81.21 | 100.17 |
| Pgls | 27.25 | 101.53 | Ppid | 40.74 | 100.15 |
| Srsf6;Srsf4 | 39.03 | 101.35 |  |  |  |

Supplementary table 2: Profilin1 is more abundant than profilin2 in NSC34 cells. Cells were treated with scr or si*Smn* and differentiated for three days. Equal amounts of cell lysate were separated by SDS-PAGE. Relative abundances of PFN1/2 were determined. LFQ values were normalized to the highest value.

| **Protein names** | **scr**  **rel. LFQ intensity** | **si*Smn***  **rel. LFQ intensity** |
| --- | --- | --- |
| Profilin1 | 92.1% | 100.0% |
| Profilin2 | 5.4% | 5.0% |

# Supplementary figures


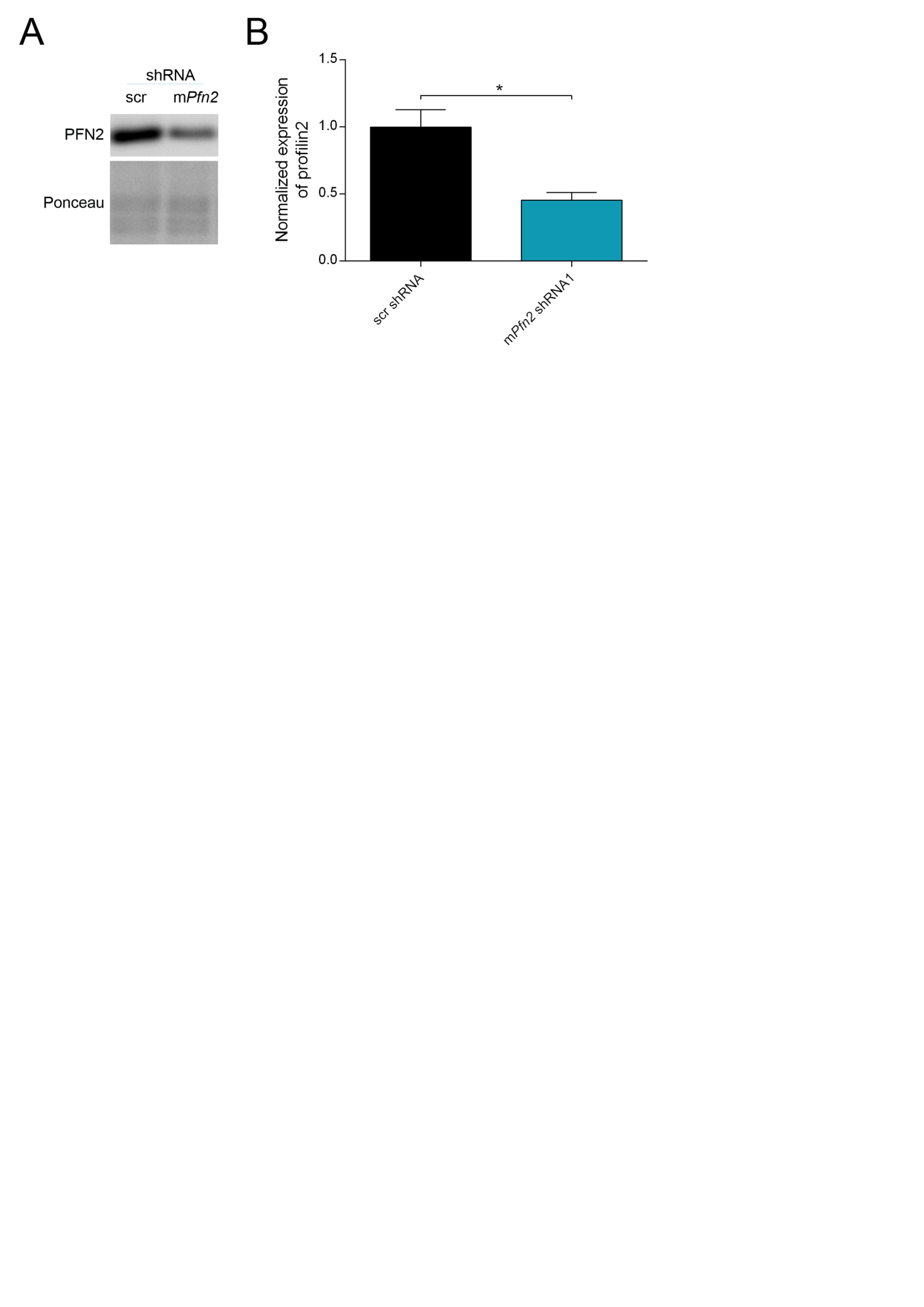


Supplementary figure 1:Profilin2 shRNA reduces protein levels by half. Cells were transfected with bicistronic plasmids containing either scr shRNA or shRNA against *Pfn2* and differentiated for three days. (A) Representative Western blot of transfected cells probed for PFN2. Ponceau was used as loading control. (B) Quantification of PFN2 levels normalized to total protein (mean ± SEM, n=3, paired two-tailed t-test, *p<0.05).


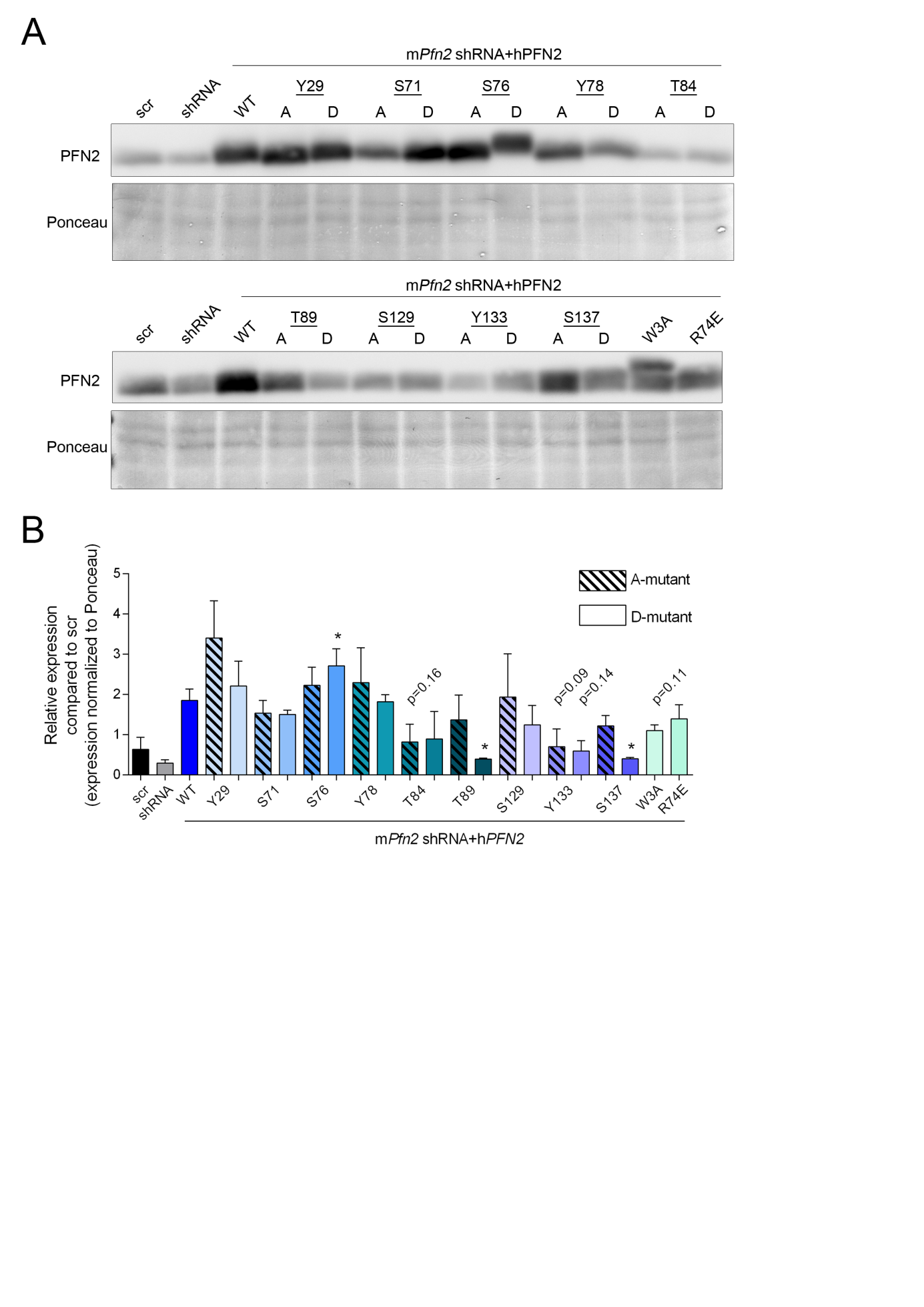


Supplementary figure 2: Differential expression pattern of profilin2 phospho-mimetics and -mutants. Cells were transfected with bicistronic plasmids containing scr shRNA or shRNA against *Pfn2* and the coding sequence of PFN2 mutants and differentiated for three days. (A) Representative Western blots of transfected cells probed for PFN2. Ponceau was used as loading control. (B) Quantification of PFN2 levels normalized to total protein (mean ± SEM, n=3, paired two-tailed t-test compared to WT, *p<0.05). Signal intensities of double bands due to differential migration of endogenous and overexpressed protein were combined. Differential posttranslational modification of profilin2 W3A may cause the distinct double bands observed for this mutant.

**Figures of uncropped blots**

**Figure 1 uncropped:** **(A-D)** Shown are the uncropped Western Blots of Figure 1A and 1D. Red boxes indicate the cropped regions.


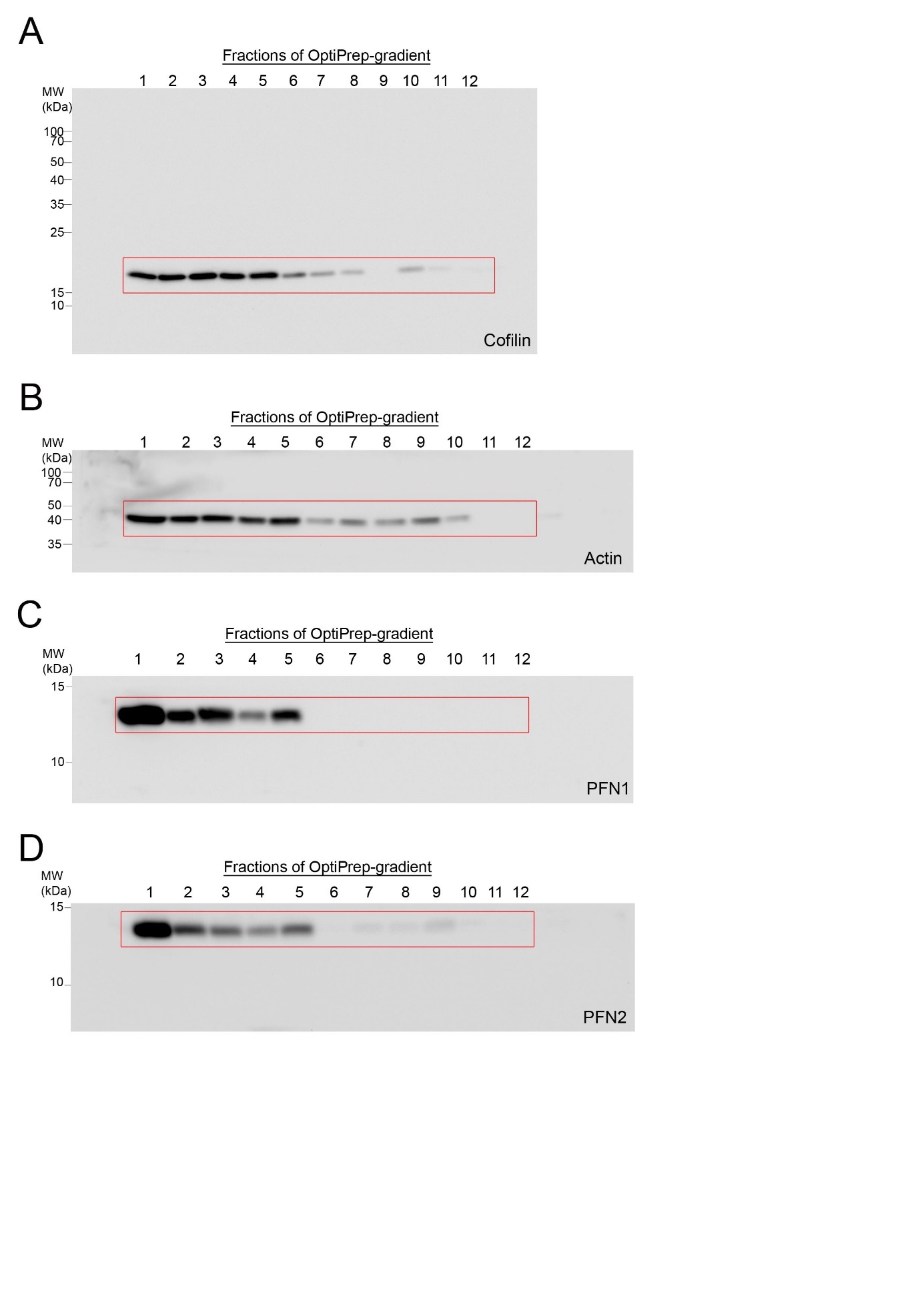


**Figure 2 uncropped:** **(A-D)** Shown are the uncropped Western Blots of Figure 2A. Red boxes indicate the cropped regions. **(C+D)** Western Blot stained for PFN2 and Ponceau stained membrane was cropped and flipped horizontally.


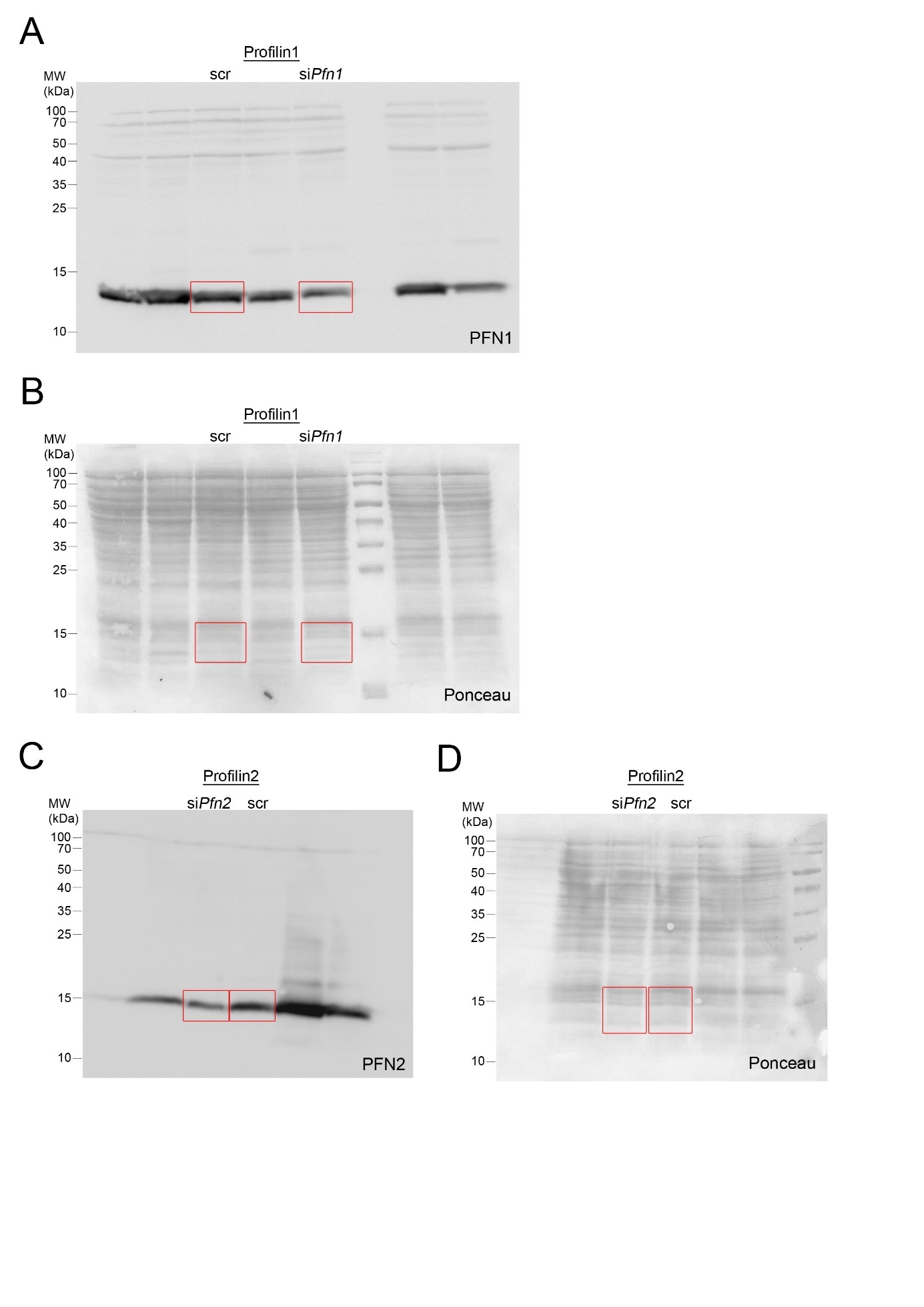


**[Figure 3 uncropped: no blots in this figure.]**

**Figure 4 uncropped: (A-C)** Shown are the uncropped Western Blots of Figure 4D. Red boxes indicate the cropped regions.


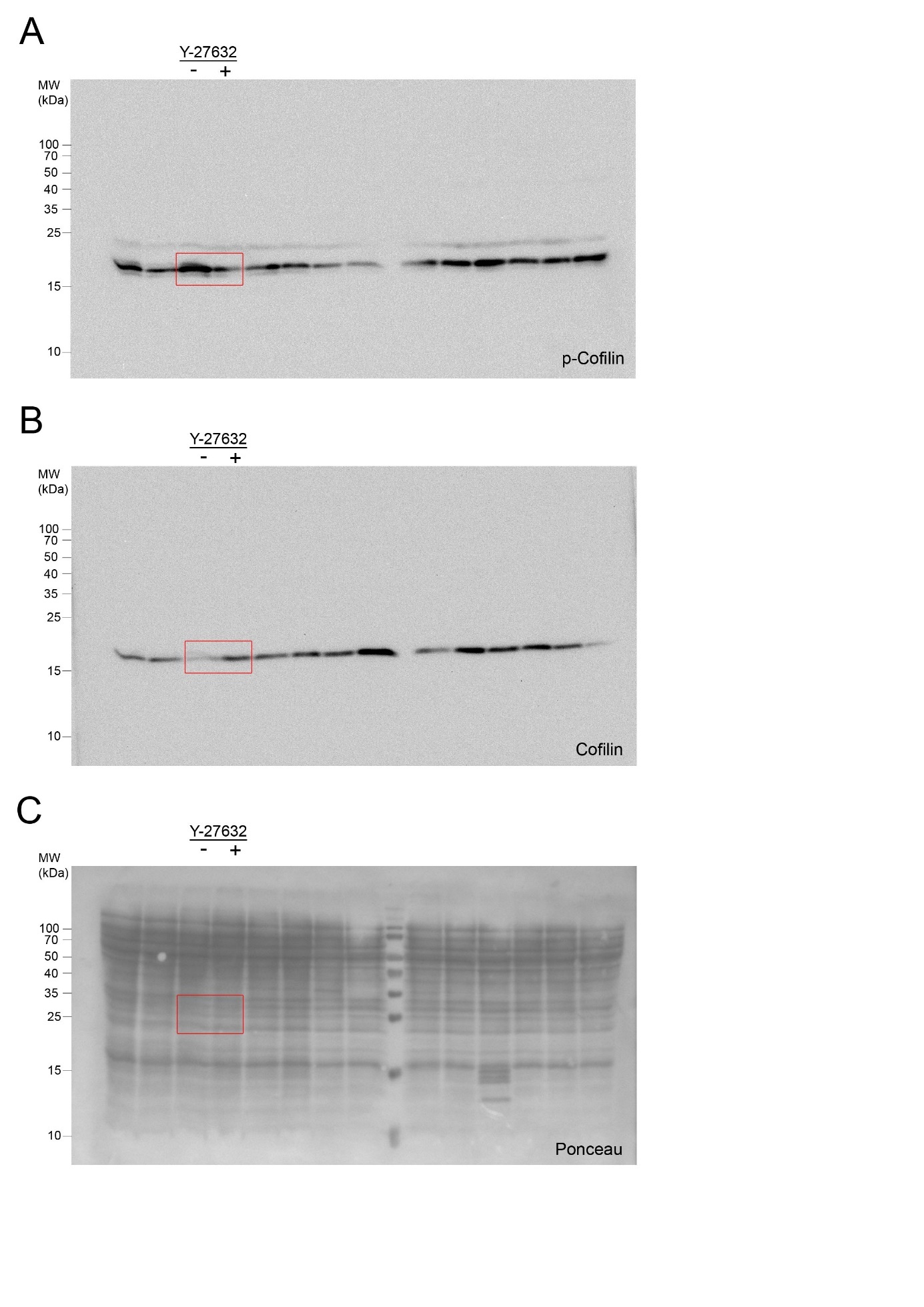


**Figure S1 uncropped:** **(A-D)** Shown are the uncropped Western Blots of Figure S1. Red boxes indicate the cropped regions.


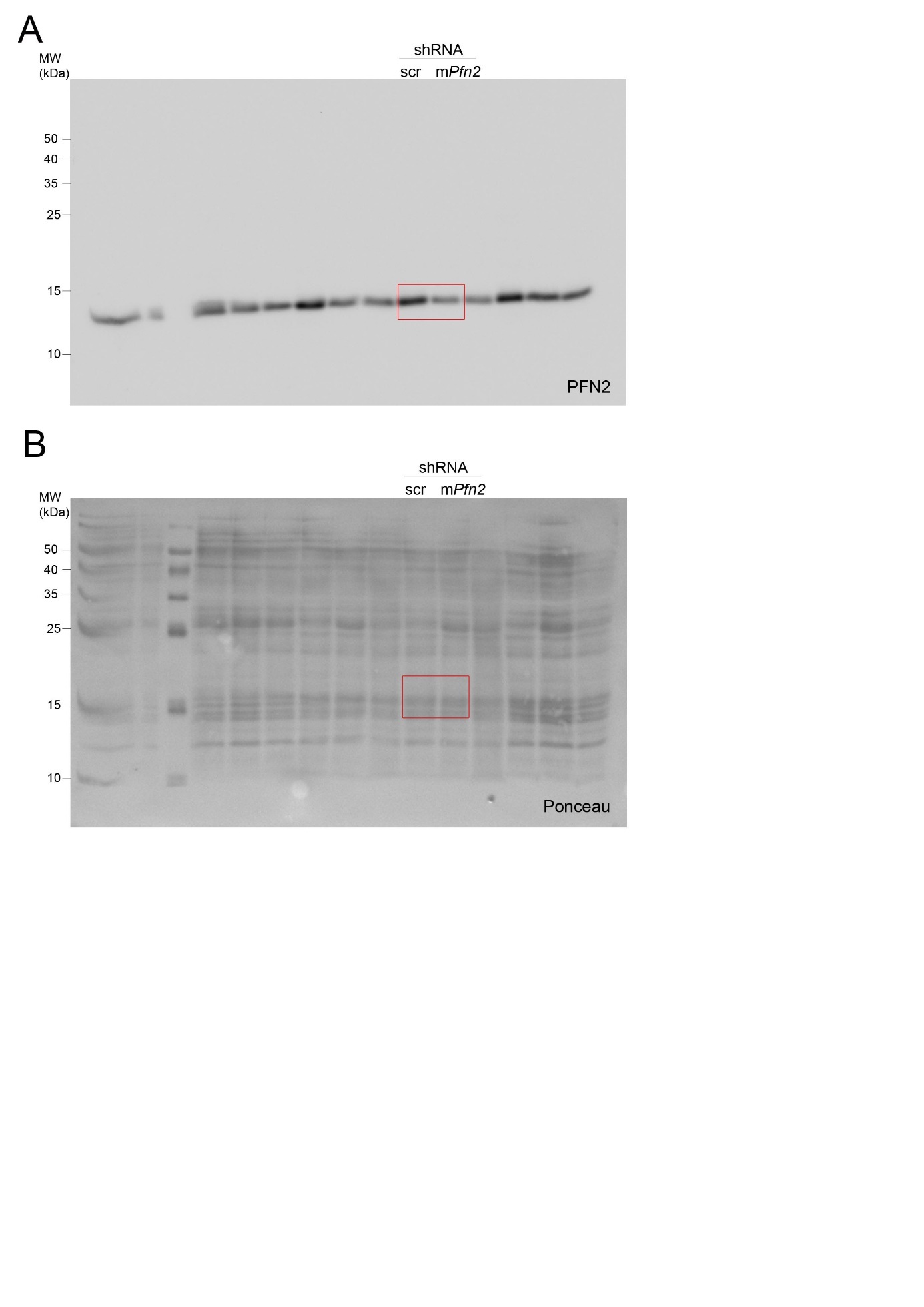


**Figure S2 uncropped:** **(A-D)** Shown are the uncropped Western Blots of Figure S2. Red boxes indicate the cropped regions.

**
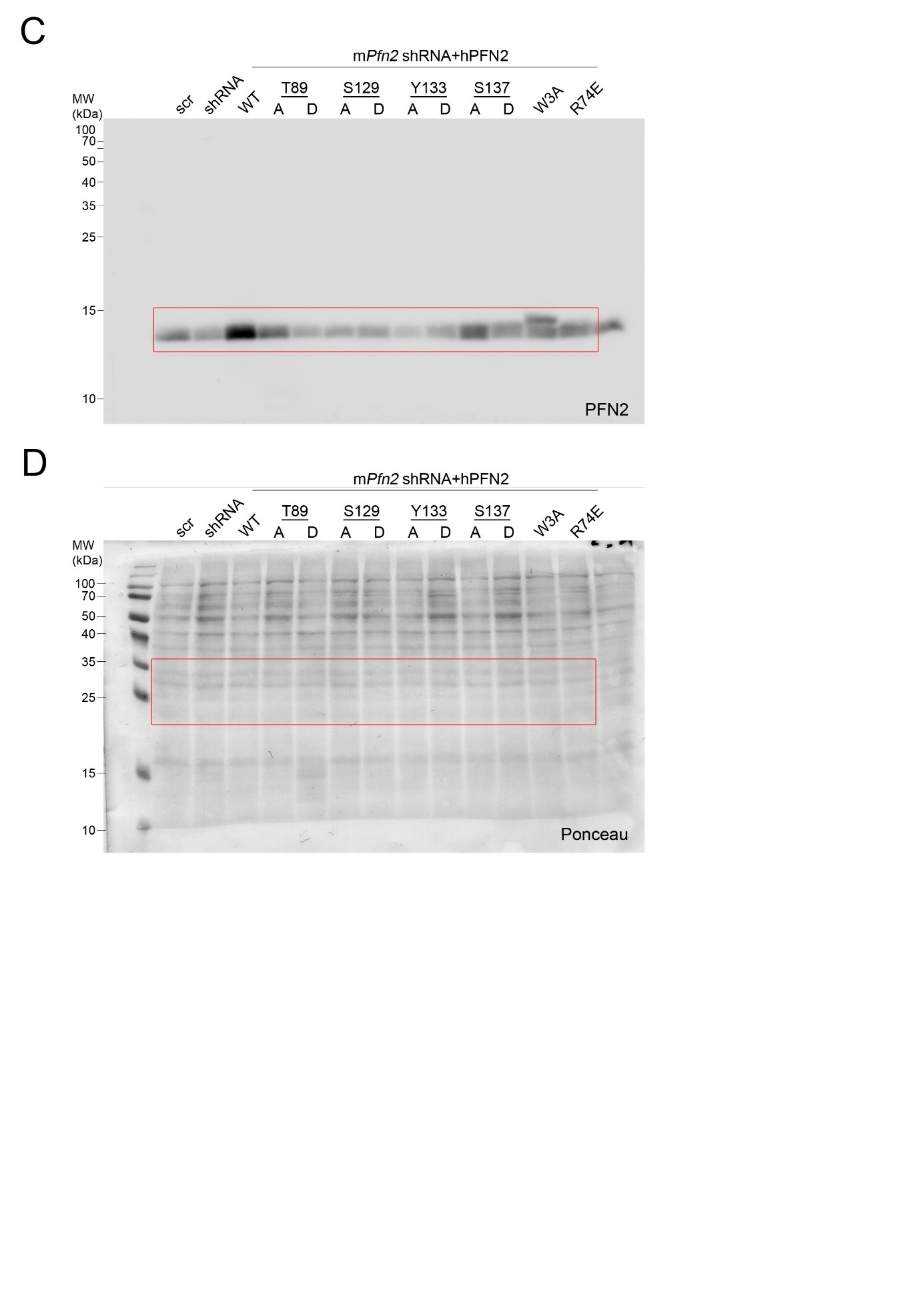
**

**Uncropped blots with visible edges**

Original blots were converted from 16 bit tif images (which have been taken for densitometry) to 8 bit images, inverted, and contrast/ brightness have both been *enhanced to demonstrate edges of the blots*. Images were finally saved as jpg files. Original file names are provided.

**Fig. 1**

170918_Rod isolation_Gradient2_Cofilin


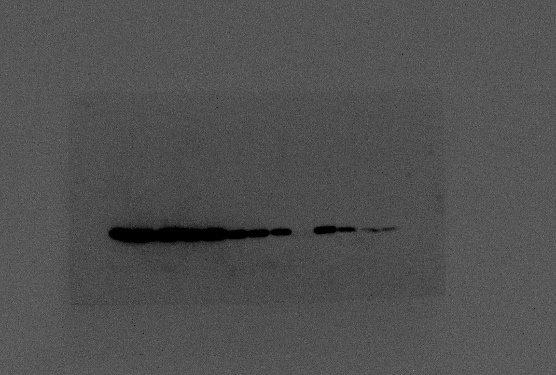


02.10.19_actin rod_actin_2min


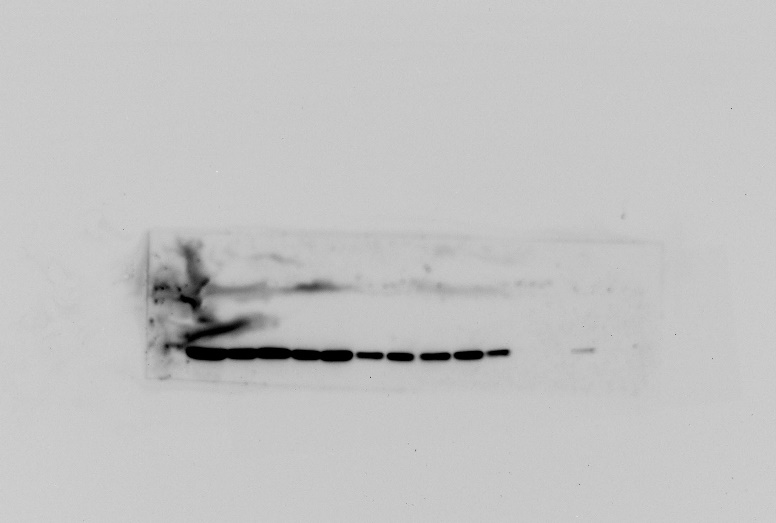


02.10.19_actin rod_PFN1_1min


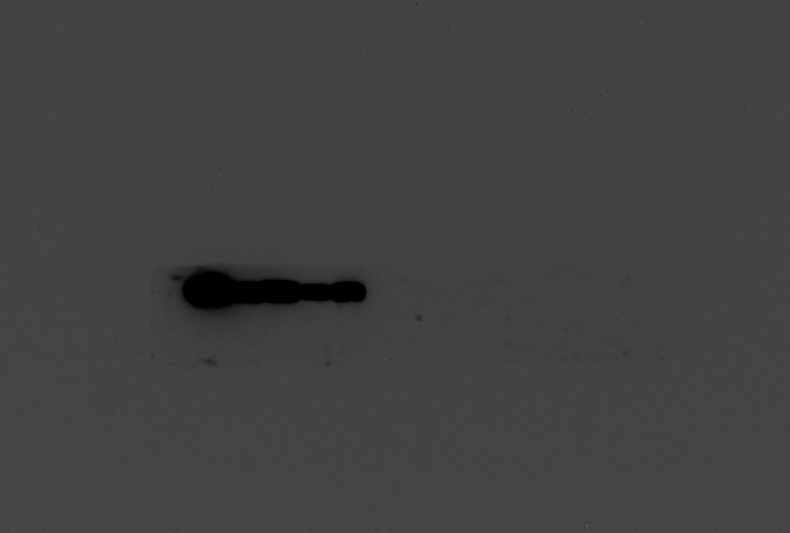


02.10.19_actin rod_PFN2_80sec


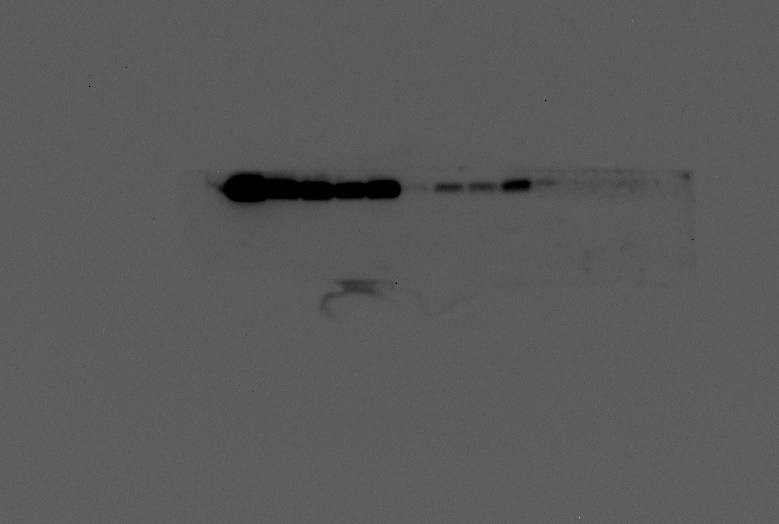


2017-Sep-27_10-31-11 MS samples rods


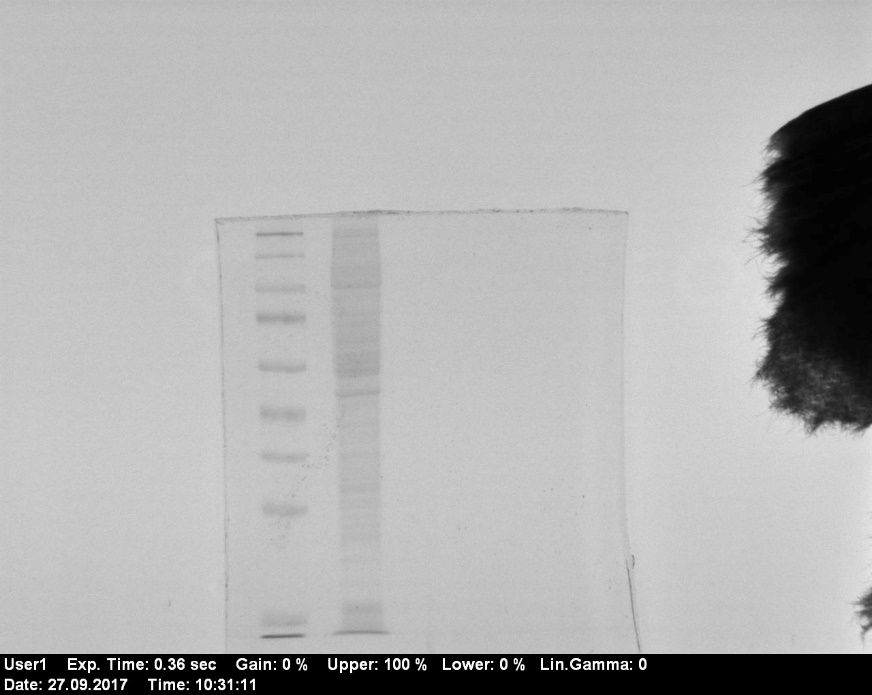


**Fig. 2**

PFN1_2sec_3.scan


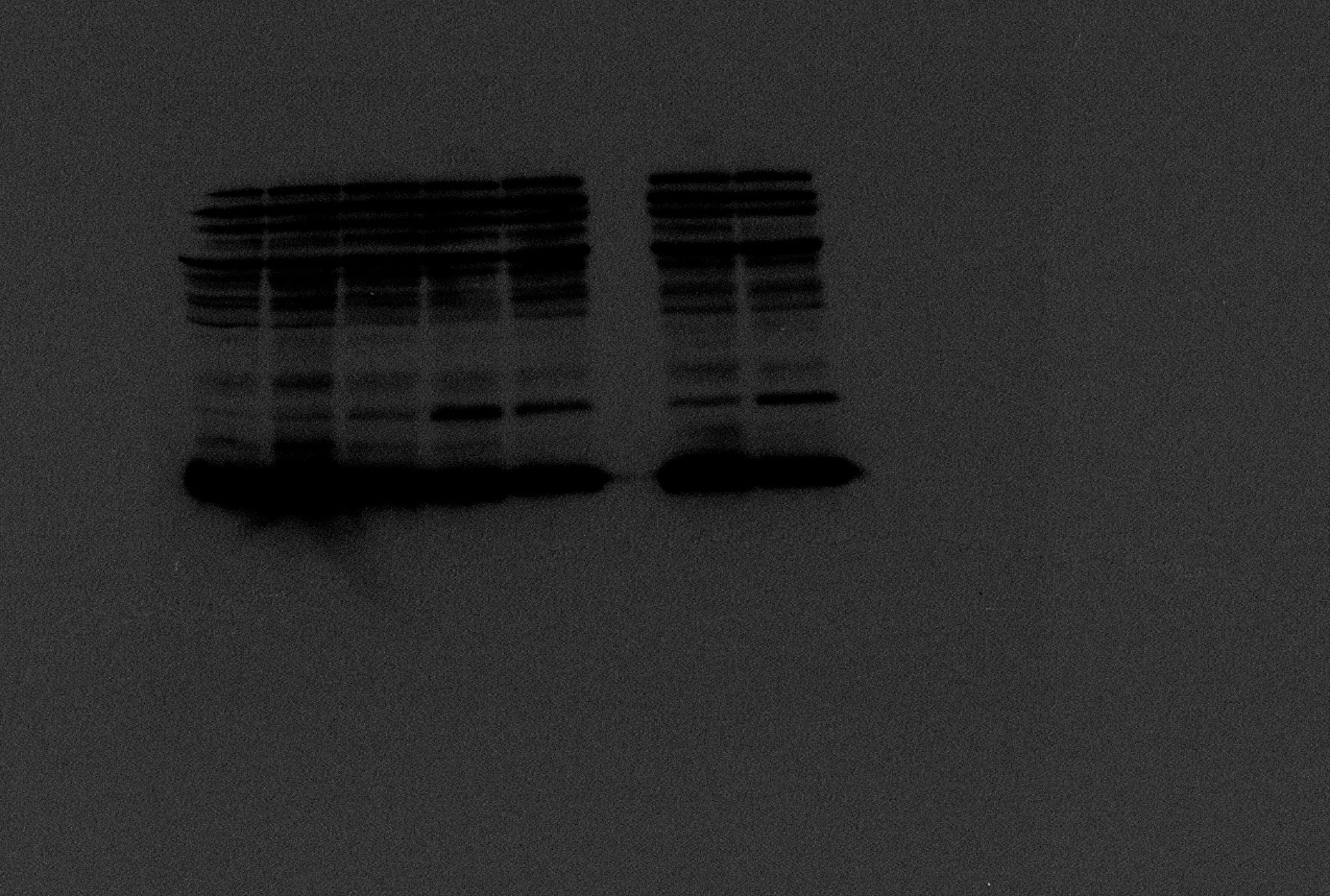


Img046


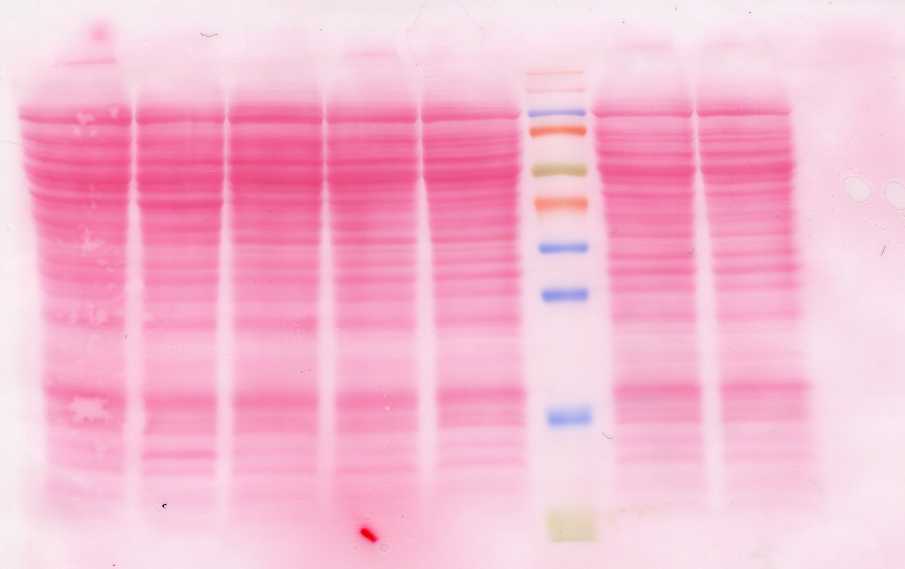


25sec


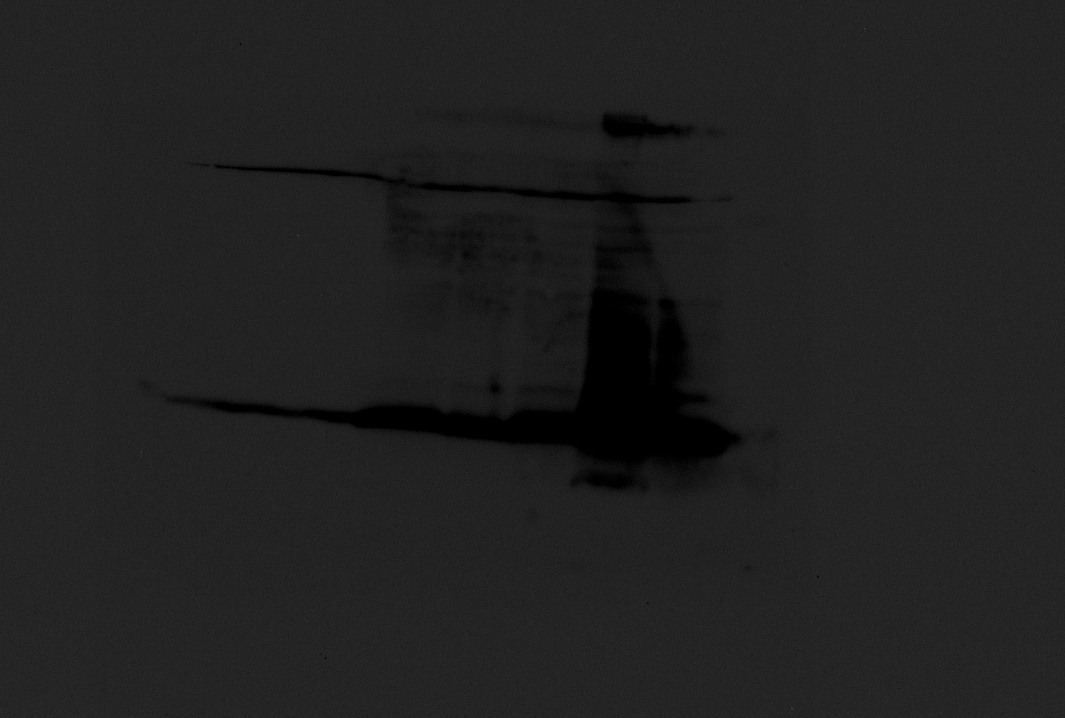


Img031


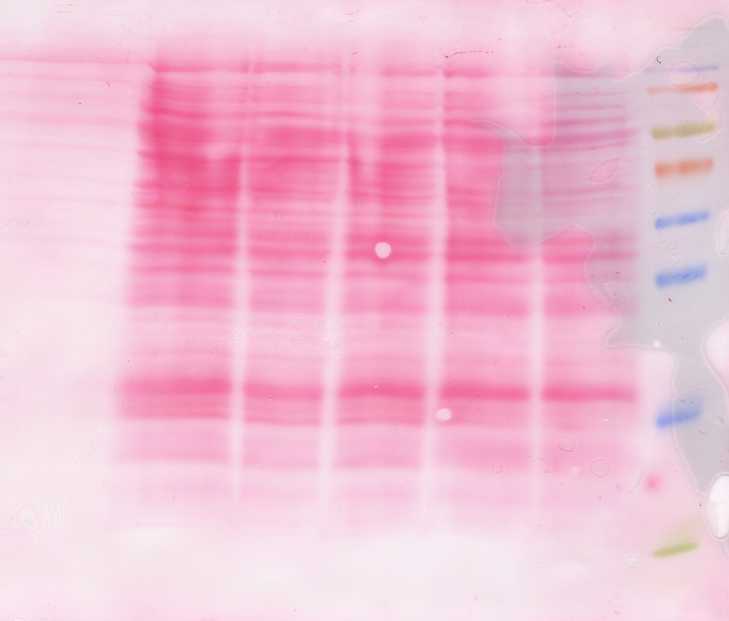


**Fig. 4**

Pcofilin [6.11.2017]


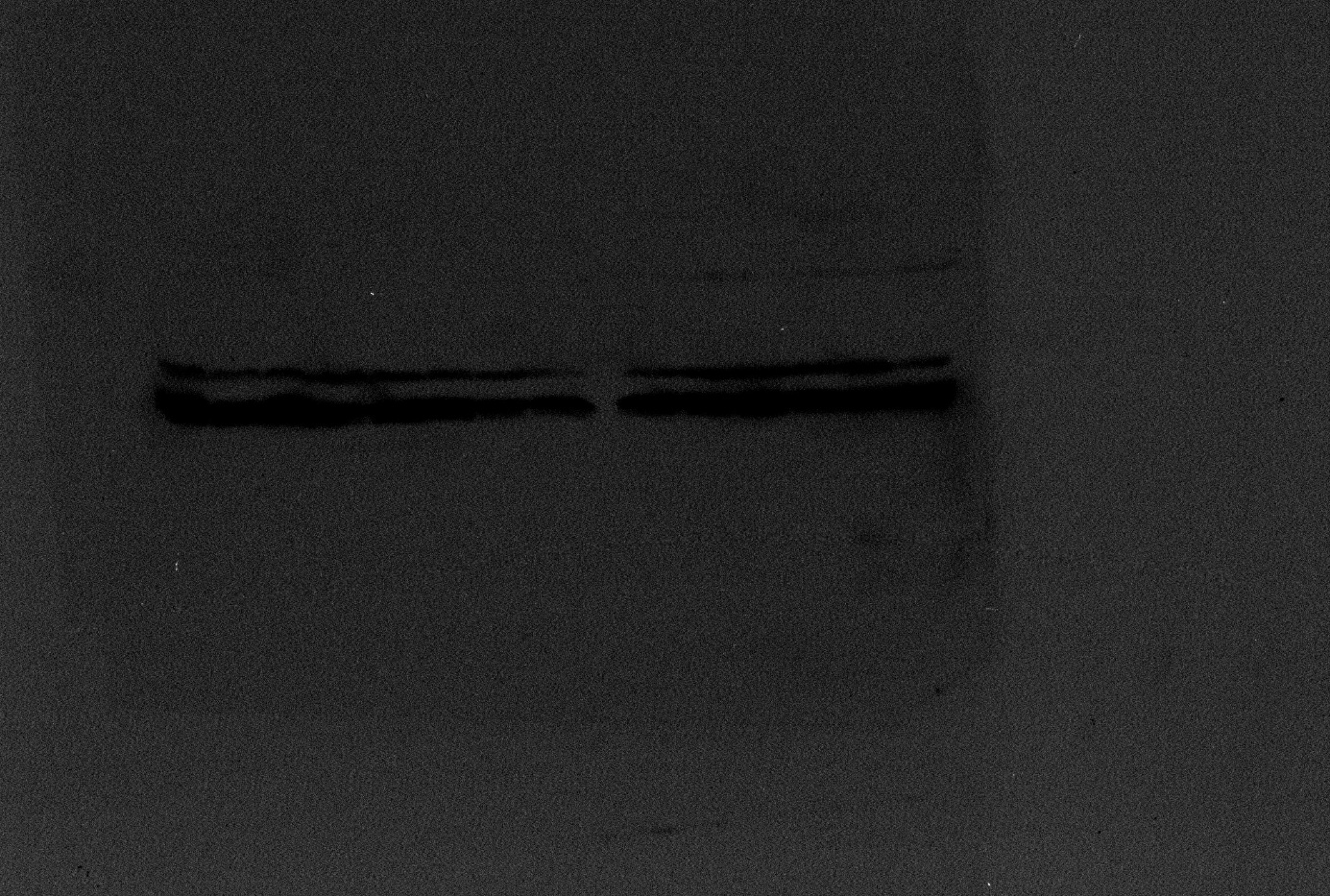


Cofilin_5min


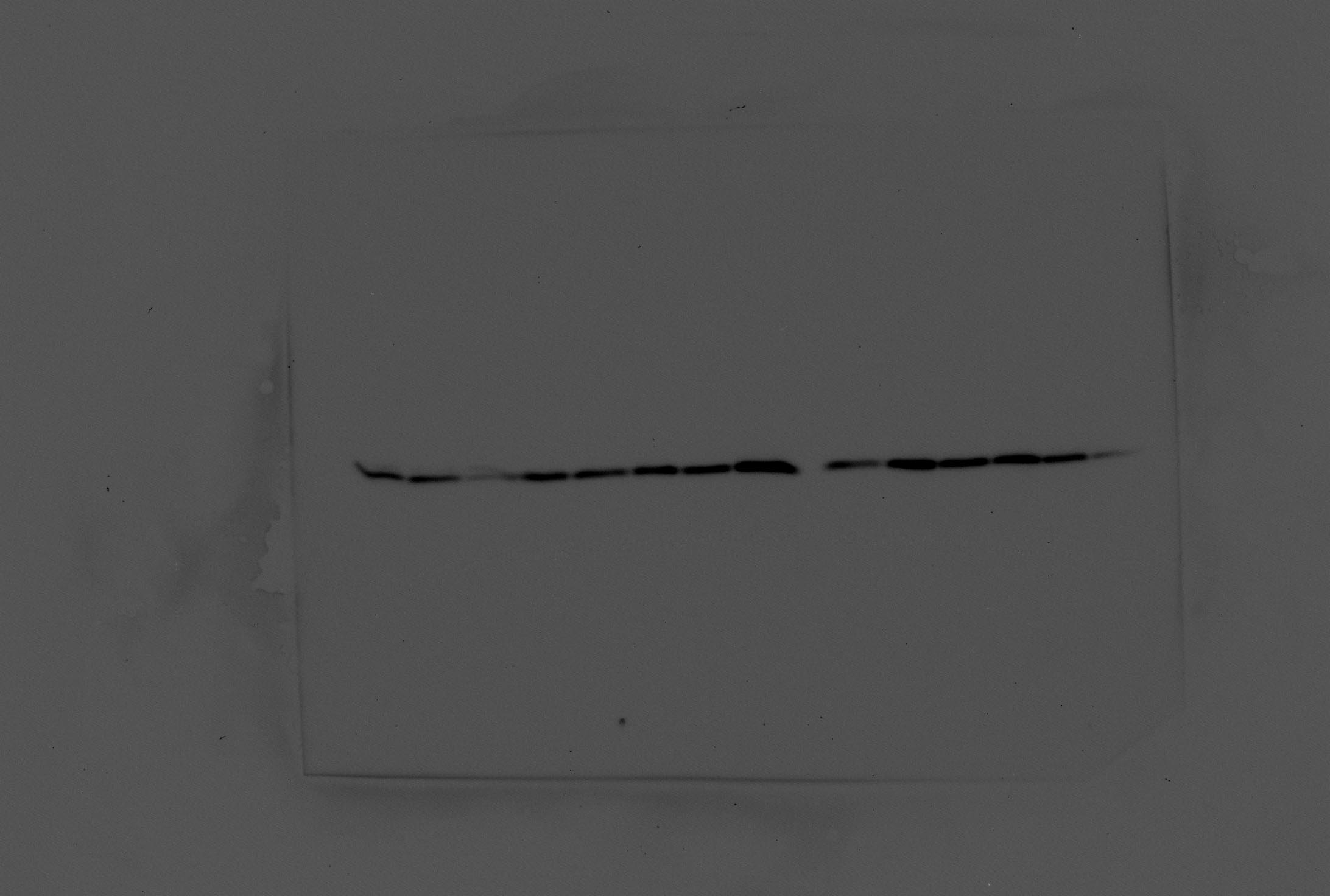


2017-Nov-03_17-07-34
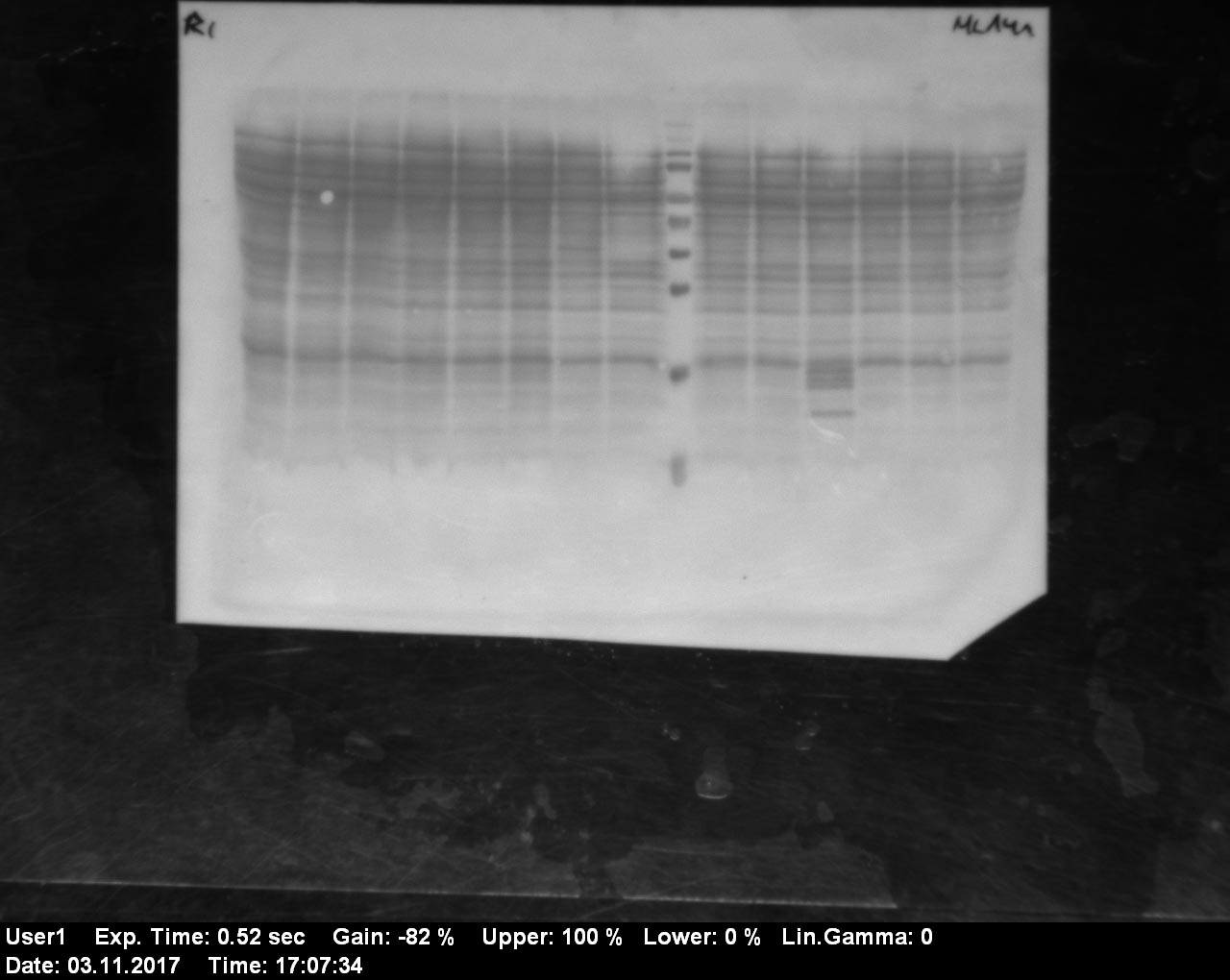


**Fig. S1**

Pfn2_2.Versuch


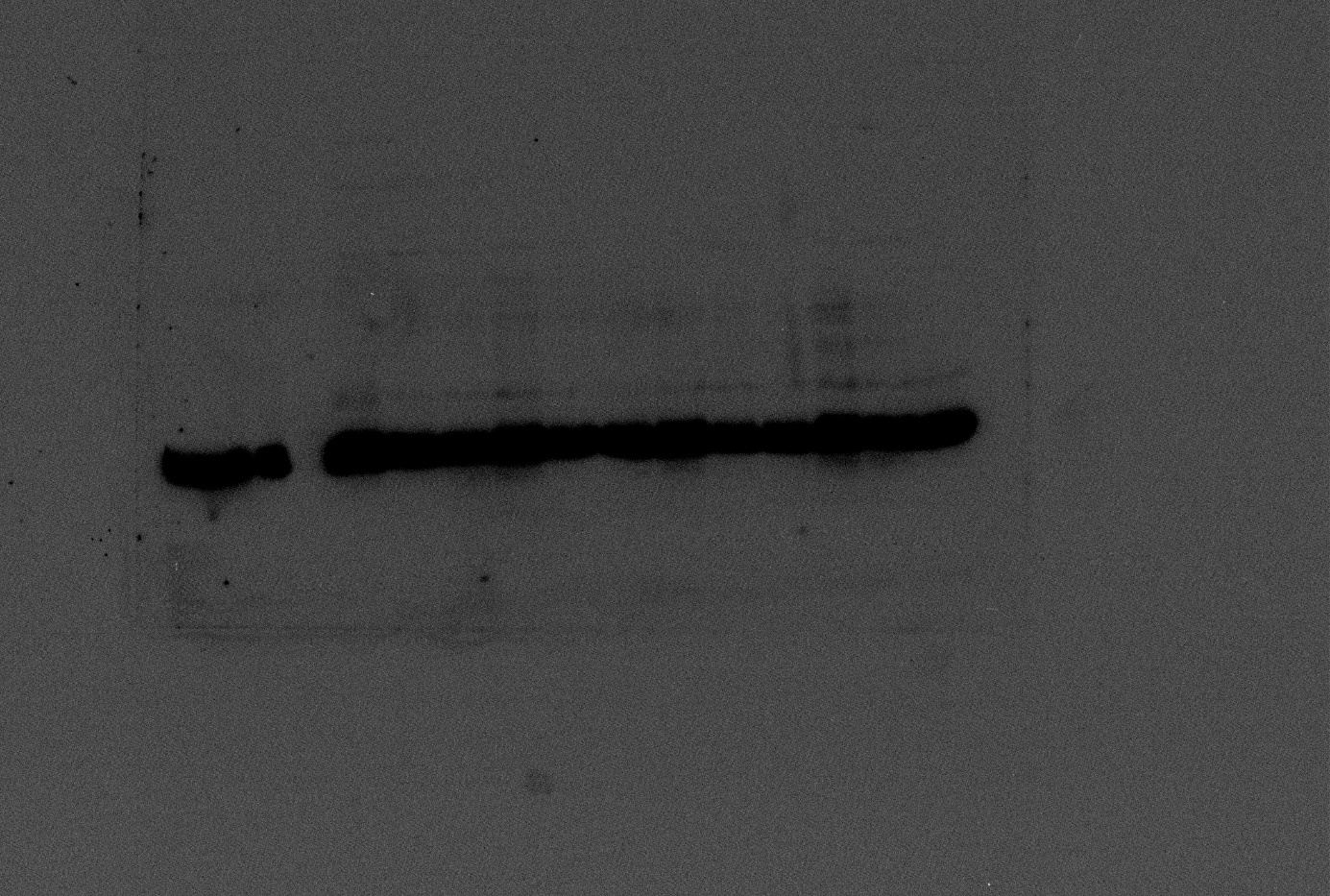


2018-Feb-20_15-36-48 Ponceau_1
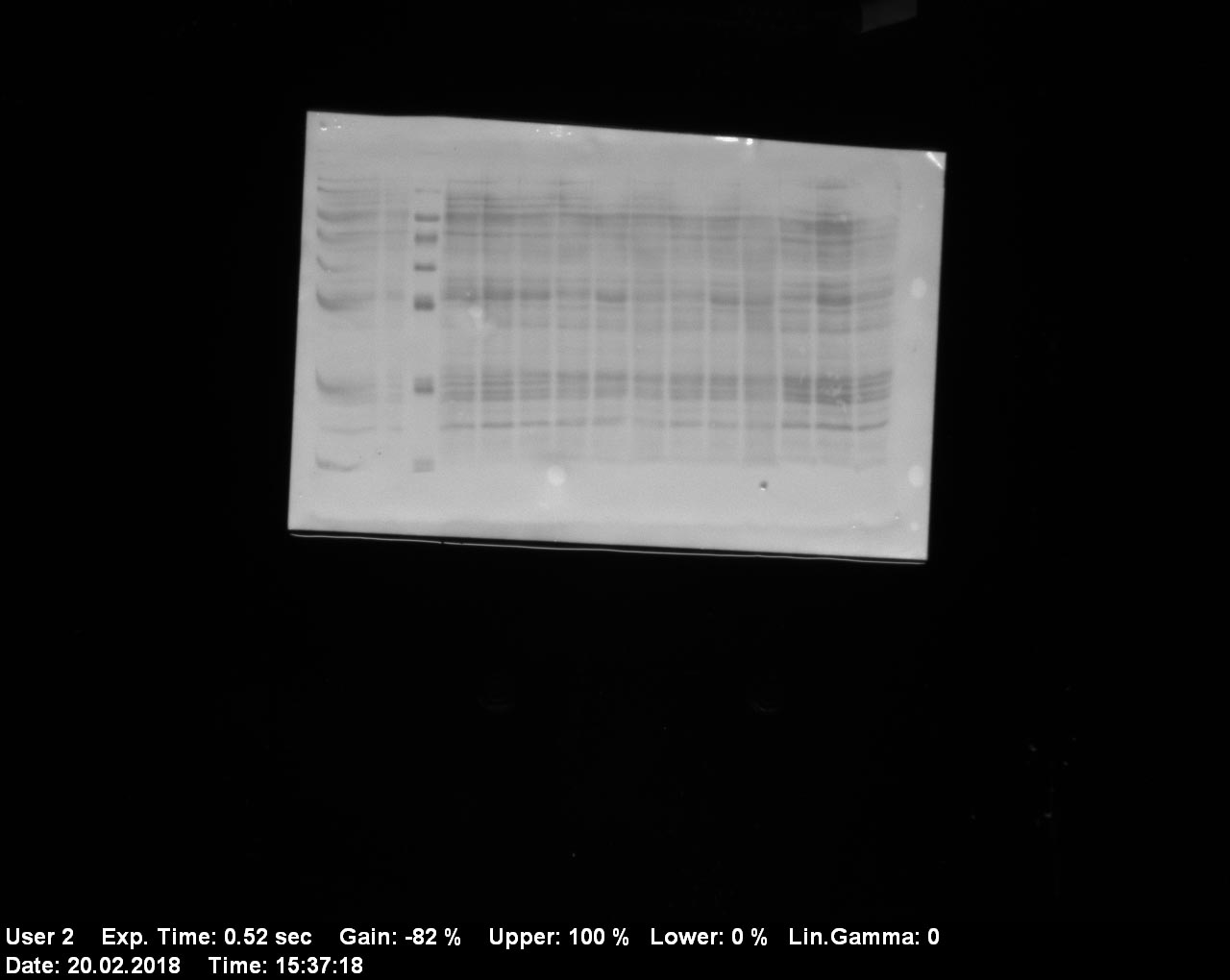


**Fig. S2**

19.09.18_1.experiment_PFN2_50sec


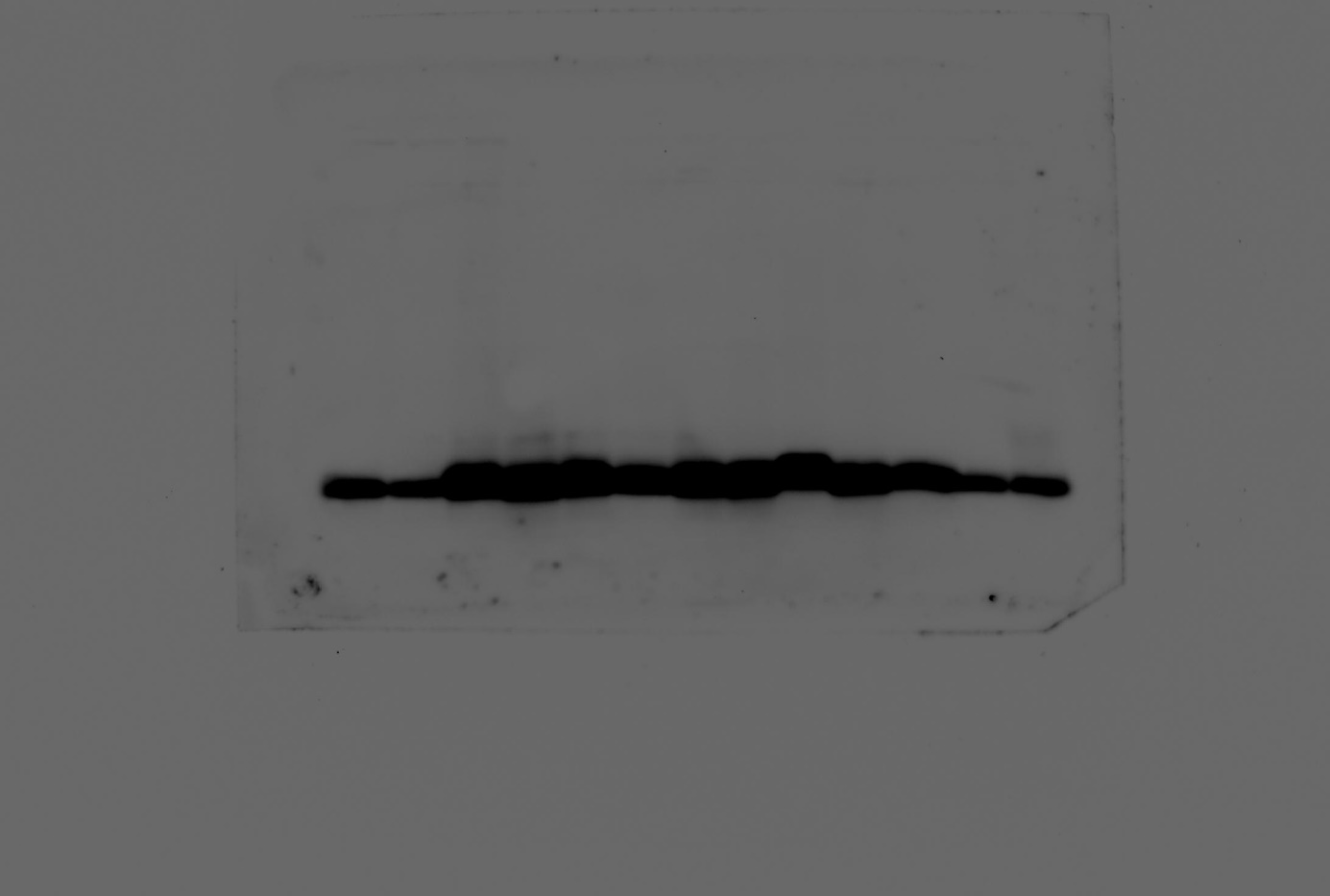


18.09.18_Ponceau_1.1


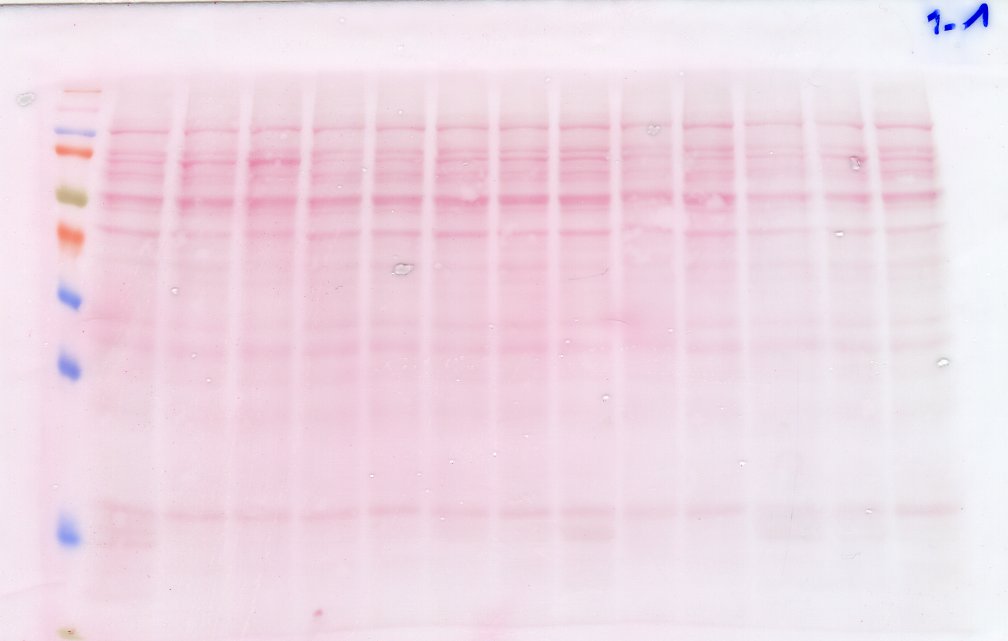


19.09.18_2.experiment_PFN2_50sec


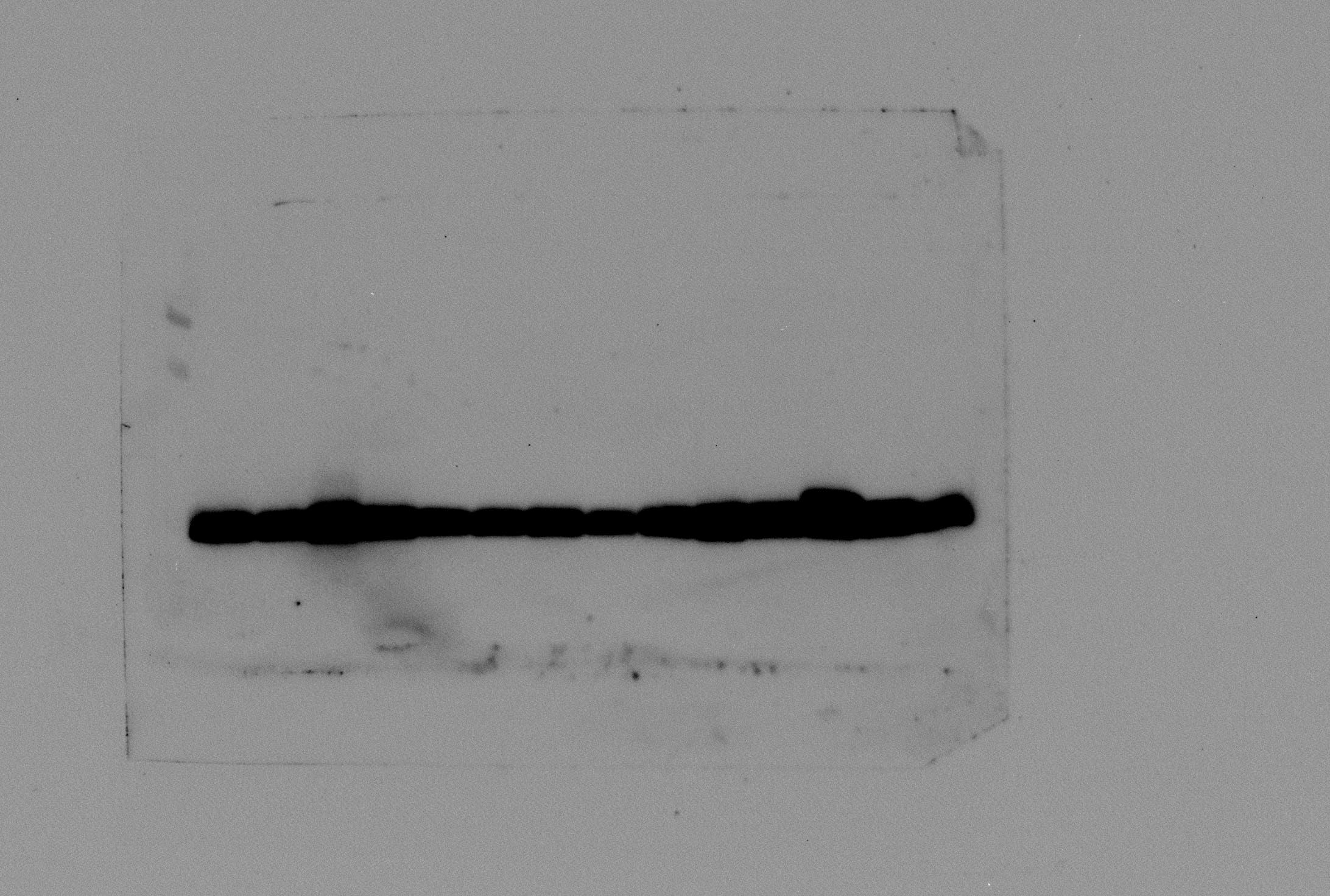


18.09.18_Ponceau_2.1


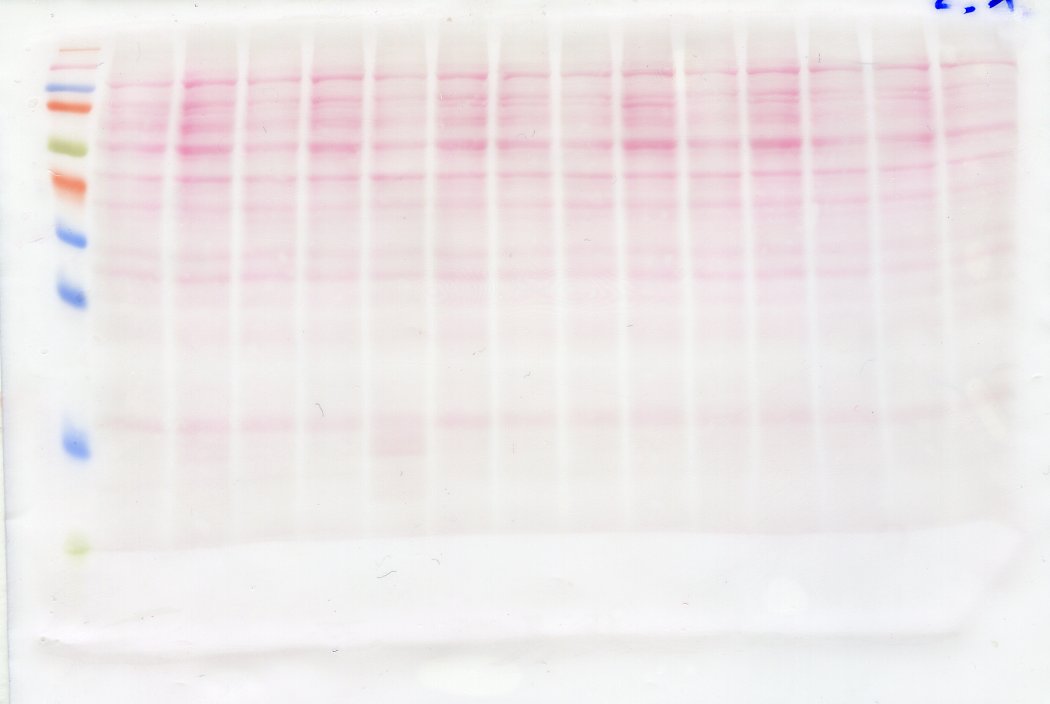

Supplement: Supplementary file 1 — Supplementary Information. [file 41598_2021_89397_MOESM1_ESM.docx]
